# Gene Profiling of Postnatal *Mfrp^rd6^* Mutant Eyes Reveals Differential Accumulation of *Prss56*, Visual Cycle and Phototransduction mRNAs

**DOI:** 10.1371/journal.pone.0110299

**Published:** 2014-10-30

**Authors:** Ramani Soundararajan, Jungyeon Won, Timothy M. Stearns, Jeremy R. Charette, Wanda L. Hicks, Gayle B. Collin, Jürgen K. Naggert, Mark P. Krebs, Patsy M. Nishina

**Affiliations:** 1 The Jackson Laboratory, Bar Harbor, Maine, United States of America; 2 National Disaster Management Institute, Seoul, Korea; Justus-Liebig-University Giessen, Germany

## Abstract

Mutations in the membrane frizzled-related protein (*MFRP/Mfrp*) gene, specifically expressed in the retinal pigment epithelium (RPE) and ciliary body, cause nanophthalmia or posterior microphthalmia with retinitis pigmentosa in humans, and photoreceptor degeneration in mice. To better understand MFRP function, microarray analysis was performed on eyes of homozygous *Mfrp^rd6^* and C57BL/6J mice at postnatal days (P) 0 and P14, prior to photoreceptor loss. Data analysis revealed no changes at P0 but significant differences in RPE and retina-specific transcripts at P14, suggesting a postnatal influence of the *Mfrp^rd6^* allele. A subset of these transcripts was validated by quantitative real-time PCR (qRT-PCR). In *Mfrp^rd6^* eyes, a significant 1.5- to 2.0-fold decrease was observed among transcripts of genes linked to retinal degeneration, including those involved in visual cycle (*Rpe65*, *Lrat, Rgr*), phototransduction (*Pde6a*, *Guca1b*, *Rgs9*), and photoreceptor disc morphogenesis (*Rpgrip1* and *Fscn2*). Levels of RPE65 were significantly decreased by 2.0-fold. Transcripts of *Prss56*, a gene associated with angle-closure glaucoma, posterior microphthalmia and myopia, were increased in *Mfrp^rd6^* eyes by 17-fold. Validation by qRT-PCR indicated a 3.5-, 14- and 70-fold accumulation of *Prss56* transcripts relative to controls at P7, P14 and P21, respectively. This trend was not observed in other RPE or photoreceptor mutant mouse models with similar disease progression, suggesting that *Prss56* upregulation is a specific attribute of the disruption of *Mfrp*. *Prss56* and *Glul in situ* hybridization directly identified Müller glia in the inner nuclear layer as the cell type expressing *Prss56*. In summary, the *Mfrp^rd6^* allele causes significant postnatal changes in transcript and protein levels in the retina and RPE. The link between *Mfrp* deficiency and *Prss56* up-regulation, together with the genetic association of human *MFRP* or *PRSS56* variants and ocular size, raises the possibility that these genes are part of a regulatory network influencing postnatal posterior eye development.

## Introduction

*MFRP* mutations in humans are associated with nanophthalmia or posterior microphthalmia with autosomal recessive retinitis pigmentosa (RP), characterized by retinal spots, foveoschisis and optic nerve head drusen [1]–[6]. A homozygous mutation of *Mfrp* in mice recapitulates central features of the human disease, including retinal spots and a slowly progressing retinal degeneration [7]–[9]. The *MFRP/Mfrp* disease phenotype is variable in humans [1]–[6] and mice [9], [10], suggesting an influence of allelic effects and/or genetic modifiers in both species. The common attributes of the human and mouse phenotypes, and the similar genetic modification on disease phenotype make *Mfrp* mutant mice attractive for delineating the mechanism(s) that underlie *MFRP/Mfrp-*associated ocular disease and its genetic variability, which are poorly understood.

Within the eye, MFRP is exclusively localized to the apical surface of the retinal pigment epithelium (RPE) and the ciliary body epithelium [11]–[13]. The protein has been suggested to play a role in the normal development as well as maintenance of photoreceptor outer segments (OS) [12]. MFRP is a type II transmembrane protein and contains multiple domains, including an N-terminal cytoplasmic domain, a transmembrane domain, two extracellular cubulin (CUB) domains, a low-density lipoprotein domain (LDLa) and a C-terminal cysteine-rich domain (CRD) [8], [14]. Complement C1q tumor necrosis factor–related protein 5 (C1QTNF5, also known as CTRP5) is expressed from the same dicistronic transcript as *Mfrp* [8], [11], [15]. Dicistronic messages often function in common pathways [16]. Therefore, it is notable that MFRP and C1QTNF5 co-localize in the posterior eye [8], [11], [15] and have been shown to interact directly by two-hybrid and biochemical studies [11]. The functional consequence of this interaction, however, is unknown, and other potentially interacting partners of MFRP remain to be identified.

To obtain insight into the functional role of MFRP in the RPE and retina during normal eye development and disease, gene-profiling studies were carried out in *Mfrp^rd6^* and wildtype postnatal eyes. Microarray analysis revealed a moderate decrease in phototransduction, visual cycle, and gene transcripts associated with retinal degeneration in *Mfrp^rd6^* mutants. Most interestingly, an increased temporal expression of *Prss56*, a gene linked to posterior microphthalmia [17] and associated with ocular growth defects in myopia [18], [19] was observed. *Prss56* upregulation appeared to be specific to *Mfrp^rd6^* mice, as it was not observed in other retinal degeneration models with similar disease progression. Broadly, our findings delineate a potential role of MFRP in postnatal development and/or maintenance of the posterior eye, and provide evidence that MFRP and PRSS56 participate in the same functional pathway.

## Materials and Methods

### Ethics Statement

Protocols using mice in this study were approved by The Jackson Laboratory Institutional Animal Care and Use Committee (Animal Welfare Assurance Number: A3268-01) in accordance with the "Guide for the Care and Use of Experimental Animals" established by the National Institutes of Health (NIH) (1996, Revised 2011).

### Animals

Mouse strains utilized in this study, B6.C3Ga-*Mfrp^rd6^/*J (*Mfrp^rd6^*) and C57BL/6J were bred and maintained in a vivarium with a 12 hour light and 12 hour dark cycle in the Research Animal Facility at The Jackson Laboratory. Autoclaved NIH31 diet (6% fat) and HCl acidified water (pH 2.8–3.2) were provided *ad libitum*.

### Gene Profiling

GeneChip Mouse Genome 430 v2.0 array (Affymetrix) was performed at The Jackson Laboratory Gene Expression Analysis Service (GES). Total RNA from *Mfrp^rd6^* and C57BL/6J eyes at P0 and P14 (three biological replicates per group) was extracted with Trizol (Life Technologies, Carlsbad, CA, USA) according to the manufacturer's instructions. Two µg of total RNA was used for cDNA synthesis with the One-Cycle Target Labeling cDNA Synthesis Kit (Affymetrix, Santa Clara, CA, USA) and fragmented cRNA was synthesized using the GeneChip IVT Labeling kit (Affymetrix, Santa Clara, CA, USA) according to the manufacturer's protocol (GeneChip Expression analysis Technical manual, Affymetrix 1999–2004). Following cRNA fragmentation, 15 µg of cRNA were hybridized on a GeneChip Mouse Genome 430 v2.0 array at 45°C for 16 h and the microarray was scanned using the GeneChip Scanner 3000 (Affymetrix, Santa Clara, CA, USA). Average signal intensities for each probe set within the arrays were calculated by and exported from Affymetrix's Expression Console (Version 1.2) software using the RMA method, which incorporates convolution background correction, sketch-quantile normalization, and summarization based on a multi-array model fit using the median polish algorithm to generate gene expression data. For this experiment, six pairwise comparisons were used to statistically resolve gene expression differences between sample groups using the R/maanova analysis package. Specifically, differentially accumulated transcripts were detected by using Fs, a modified F-statistic incorporating shrinkage estimate of variance components from within R/maanova. Statistical significance levels of the pairwise comparisons were calculated by permutation analysis (1000 permutations) and adjusted for multiple testing using the false discovery rate (FDR) *q*-value threshold of 0.05. For each probe set, the raw intensities for all probes were log2-transformed. The log2-transformed intensities were quantile-normalized and a volcano plot was generated for each of the six pairwise comparisons. Differentially accumulated transcripts were classified into various biological pathways using the Ingenuity Pathway Analysis Systems software (Ingenuity Systems, Qiagen, Valencia, CA, USA). Microarray data (MIAME compliant) were submitted to the Gene expression Omnibus (GEO) database (http://www.ncbi.nlm.noh.gov/geo) under GEO Accession Number GSE53411 (http://www.ncbi.nlm.nih.gov/geo/info/linking.html).

### Ingenuity Pathway Analysis

Ingenuity pathway analysis software (IPA; Ingenuity Systems, www.ingenuity.com, Summer 2013 release, Qiagen, Valencia, CA, USA) was used to identify potential networks affected by the disruption of *Mfrp* across one dataset (*Mfrp^rd6^* versus C57BL/6J at P14). The dataset was derived from differential gene expression analysis resulting from the comparison of mRNA from whole eye tissue samples of *Mfrp^rd6^* versus C57BL/6J mice. Data uploaded into IPA consisted of Affymetrix Mouse Gene 430 2.0 probe sets as identifiers. Each identifier was mapped to its corresponding object in the IPA knowledge base (Summer 2013 release). Expression results were limited to genes having a *q*-value <0.05. Gene networks were algorithmically generated based on their connectivity to the uploaded data set. Networks pertaining to phototransduction and visual pathways were retained, and expression and significance values were overlaid onto networks of interest so as to identify differential patterns of up- and down-regulated genes. In each network, molecules are represented as nodes, and the biological relationship between two nodes is represented as an edge (line). All edges are supported by at least one reference from the literature, from a textbook, or from canonical information stored in the IPA Knowledge Base. The intensity of the node color indicates the degree of up- (red) or down- (green) regulation with regards to all expression values in the dataset. Nodes are displayed using various shapes that represent the functional class of the gene product; these are defined in the figure legends. Edges are displayed with various labels that describe the nature of the relationship between the nodes (e.g., P for phosphorylation, T for transcription).

### Isolation of RPE and Retina

Mice were sacrificed by carbon dioxide asphyxiation and enucleated eyes were placed in chilled phosphate buffered saline (PBS). The connective tissue, muscles and conjunctiva were carefully removed using iris scissors. A circumferential incision was made below the level of ciliary body and the anterior segment consisting of cornea, lens, iris and ciliary body was discarded. The neural retina was peeled from the RPE layer using surgical forceps and the remaining posterior eyecup consisting of the sclera, choroid and RPE was flash frozen in liquid nitrogen and stored at −70°C.

### Quantitative Real time PCR

Eyes were collected at different time points (P7, P14 and P21). Total RNA was extracted from whole eyes, retina, and RPE cells using Trizol reagent (Life Technologies, Carlsbad, CA, USA) combined with the RNeasy kit (Qiagen, Valencia, CA, USA) as per manufacturer's instructions. Genomic DNA contamination of RNA was prevented by on-column treatment with DNase I (Qiagen, Valencia, CA, USA) according to the manufacturer's instructions (Qiagen, Valencia, CA, USA). One µg of total RNA was reverse transcribed using the Retroscript kit (Ambion, Life Technologies, Carlsbad, CA, USA). Quantitative real-time PCR (qRT-PCR) was performed with the SYBR Green Master Mix (Life Technologies, Carlsbad, CA, USA) and gene-specific primers using the ViiA7 Real Time PCR Cycler (Life Technologies, Carlsbad, CA, USA). Appropriate controls including no RT control was included in the assay to rule out genomic DNA contamination of the cDNA samples. A relative fold change in gene transcript was calculated using ViiA7 software V1.2.2 (Life Technologies), applying the comparative CT method (ΔΔC_T_) and was quantified using 2^-ΔΔ*C*^_T_ with β-actin as an internal calibrator. Melting curve analysis was performed to validate accurate amplification of the target gene. The primers used for qRT-PCR were designed using mouse qPrimerDepot (mouseprimerdepot.nci.nih.gov). The primers spanned exon-exon borders that overlapped intron(s) (Table 1).

**Table 1:** Primers for qRT-PCR.

Gene	Forward primer (5′ to 3′)	Reverse primer (5′ to 3′)	Amplicon size (bp)
*Prss56*	GTTTGACCCGCAGACTTTTC	ACCCTGGGGAAGGCAAAT	100
*Rpe65*	GATGGCTTGAAACGATCACTG	GATCCCTCCACTGAAAGCAG	90
*Lrat*	CTAATCCCAAGACAGCCGAA	TATGGCTCTCGGATCAGTCC	103
*Rgr*	AGGTACAGGAGGGCATAGGG	TACCGGTTCATGGAGCAGA	110
*Rgs9*	GTTCTGCATGTCCTTCACCA	GAATTCATCCAGGGTCCAGA	107
*Fscn2*	TTCATCCTGATTGGCTGAG	AACTCTTCGACCTGGAGCAA	107
*Guca1b*	CCAGGAAGTCAATGGTGTTG	GTTCAAGCGCTTCTTCAAGG	107
*Pde6a*	GCCACCTTGCTCTGTACCT	CATGATGCTGGAGCAGACAC	105
*Rpgrip1*	GAATCAGCTCCACGTTCTCC	CATCCAAAGTTGAAAAGCCTG	99
*Actb*	CCAGTTCGCCATGGATGACGATAT	GTCAGGATACCTCTCTTGCTCTG	207

### Western blot analysis

Whole eyes were dissected from C57BL/6J and *Mfrp^rd6^* mice and placed in ice-cold PBS. The anterior portion of the eye including cornea, lens and ciliary body was excised. The neural retina was gently peeled from the underlying RPE layer, and the RPE layer including the choroid and sclera was flash frozen in liquid nitrogen and stored at −70°C. For preparation of tissue lysates, the aggregate RPE sample was combined with 100 µl of 1× SDS Laemmli sample buffer (Bio-Rad Laboratories, Hercules, CA, USA) containing 5% β-mercaptoethanol, and sonicated three times for 5 min using a probe sonicator 3000 Ultrasonic Liquid Processor (Misonix Incorporated, Farmingdale, NY, USA) and stored in a −70°C freezer. The protein samples were suspended using a vortex mixer. Proteins were resolved by SDS-PAGE using a 10% Tris-glycine gel, (Bio-Rad Laboratories, Hercules, CA, USA) and transferred onto nitrocellulose (Bio-Rad Laboratories, Hercules, CA, USA) by semi-dry electroblotting using the Trans Blot Turbo Transfer System (Bio-Rad Laboratories, Hercules, CA, USA) according to the manufacturer's instructions. The membrane was washed briefly in PBS and blocked with Odyssey blocking buffer, (927–40000; LI-COR Biosciences, Lincoln, NB, USA) for 1 h at room temperature and subsequently incubated with anti-RPE65 antibody (ab13826; Abcam, Cambridge, MA, USA) at 1∶1000 dilution, overnight at 4°C. After four 5-min washes in 1× TBS containing 0.05% Tween20 (TBST), the blots were incubated with secondary antibodies, anti-mouse IRDye 700DX conjugated antibody (KFA011; Rockland Immunochemicals Inc, Gilbertsville, PA, USA) in blocking buffer for 45 min. Following four washes for 5 min in 1× TBST, the blots were scanned using the Odyssey Infrared Imaging system (LI-COR Biosciences, Lincoln, Nebraska, USA) at 700 nm. Blots were stripped and incubated with mouse β-actin antibody (A5441; Sigma, St. Louis, MO, USA) at a 1∶10,000 dilution overnight at 4°C and processed as previously described. Odyssey Infrared Imaging System software 3.0 was used to quantify the protein bands using a standard curve after normalizing with the β-actin control. The normalized, relative change in protein concentration was expressed in arbitrary units.

### *In situ* hybridization

Eye tissue from P14 C57BL/6J and homozygous *Mfrp^rd6^* mice was freshly harvested and fixed in 4% paraformaldehyde for 24 h at 4°C, immersed in 70% ethanol, dehydrated and embedded in paraffin blocks [12]. Five µm sections were cut and 3–4 sections were placed on positively charged glass slides (Milennium 2000 superfrost Adhesive slides, StatLab, McCkiney, TX, USA), which were then baked at 60°C for 1 h and stored at −20°C prior to *in situ* hybridization. We performed *in situ* hybridization using both the QuantiGene ViewRNA ISH Tissue 1-Plex and 2-Plex assay according to the manufacturer's instructions (Affymetrix, Santa Clara, CA, USA). Briefly, PFA- fixed paraffin-embedded tissues were baked, dewaxed using xylene for 5 min with gentle agitation, twice, followed by two ethanol washes for 5 min each. A hydrophobic barrier was drawn on the glass slide using a template and the samples were heated at 90–95°C for 10 min, followed by protease treatment at 40°C using a Thermobrite Controlled Temperature Slide Processing System (Abbott Laboratories, Abbott Park, IL, USA) for 10 min. Both heat and protease treatments were optimized for the eye tissue and to allow probe to access the specific RNA by unmasking the RNA. Mouse *Prss56* Type 1 (FastRed) probe set (VB1-15207) and Mouse Glul Type 6 probe (FastBlue) set (VB6-16850) were custom designed (Affymetrix, Santa Clara, CA, USA) for target hybridization. Each probe set contained 20 oligonucleotide pairs that were specific for *Prss56* or *Glul* and had a binding site for TYPE-specific sequences for signal amplification. Samples were incubated with *Prss56* probe and/or *Glul* probe or no probe control at 40°C for 3 h using the slide processer. For signal amplification, a series of sequential hybridization steps were carried out using bDNA technology and signals were detected by addition of Fast Red substrate and/or Fast Blue substrate to the tissue section as per manufacturer's instructions (Affymetrix, Santa Clara, CA, USA). Target RNA was detected at specific sites within the tissue by chromogenic substrate deposition, which was visualized by using an SP5 confocal microscope (Leica Microsystems Inc, Buffalo Grove, IL, USA) and fluorescent microscopy (Leica Microsystems Inc). Nuclei were visualized with Vectashield mounting medium with DAPI (4′,6-Diamidino-2-Phenylindole, Dihydrochloride) (H1200, Vector Laboratories Inc, Burlingam, CA, USA). For FastRed substrate, we imaged using Cy3 filter and for FastBlue substrate, we imaged using the Cy5 filter. Images were processed using the Image J software (NIH, Bethesda, MA, USA) and Adobe Photoshop CS6 (Adobe Systems Incorporated, San Jose, CA, USA) was used to create final images.

### Histological analysis and Immunofluorescence

The procedure for histological assays was previously described [20]. Briefly, the mice were euthanized by carbon dioxide asphyxiation. The harvested whole eyes were placed in either 4% paraformaldehyde or ice-cold acetic acid/methanol solution overnight followed by paraffin embedding using standard protocol. Eyes were cut into 6 µm sections, stained using Hematoxylin & Eosin, and visualized by light microscopy. For Immunofluorescence, PFA fixed sections were used. Deparaffinized sections were incubated with Mouse anti-Glutamine monoclonal antibody (MAB302, Chemicon) at a dilution of 1∶200 overnight at 4°C. Glutamine synthetase was fluorescently labeled using Donkey Anti-mouse Alexa 488 (A21202, Life technology) at a dilution of 1∶200 at RT for 1 h and visualized by fluorescent microscopy (Leica Microsystems Inc).

### Statistical Analysis

In this study, GraphPad Prism Version 5 software was used for statistical analyses. Student's t-test was used to calculate statistical significance (*p-*value) between two groups. *P*-values <0.05 were considered statistically significant.

## Results

### Microarray analysis of *Mfrp^rd6^* mutant mice relative to controls

In *Mfrp^rd6^* mutant mice, the earliest phenotypic changes, observed at P14, are disorganization and shortening of OSs [7], [12]. The absence of MFRP in *Mfrp^rd6^* RPE cells appears to cause the OSs to develop abnormally, and, combined with the observed *in vivo* impairment of RPE phagocytosis [12] leads in turn to progressive retinal degeneration. MFRP, therefore, plays a central role in the normal function of RPE, which is essential for photoreceptor maintenance. To determine the functional role of MFRP in the eye, gene expression profiling of homozygous *Mfrp^rd6^* and C57BL/6J eyes was carried out at P0 and P14. Strain C57BL/6J served as control, as the *Mfrp^rd6^* allele has been introgressed for more than seven generations on this background and genome-wide single nucleotide polymorphism analysis indicates at least 95% shared identity between B6.C3Ga-*Mfrprd6/*J and C57BL/6J [10].

**Figure 1:**
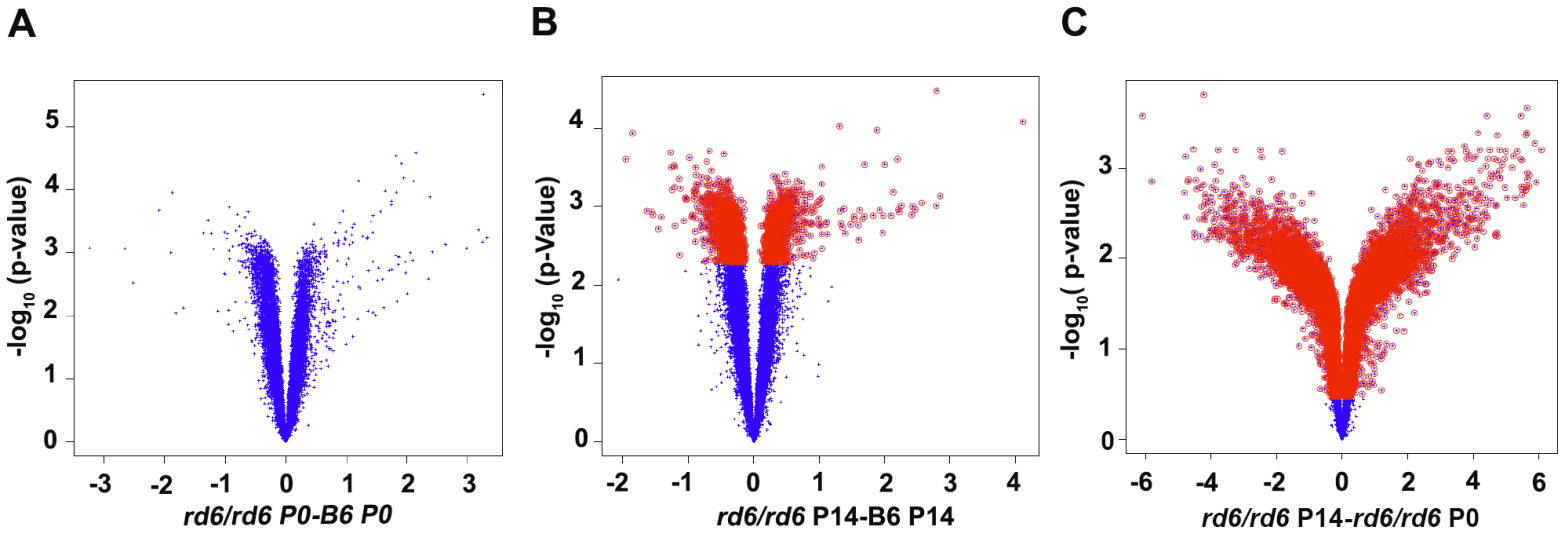
Volcano plots showing the relationship between fold change (represented as mean A – mean B) and the level of significance (represented by the *Fs* permutated *p*-value). Differentially expressed probe sets (q<0.05 shown in red across all fold change levels) at and fold change greater than 2 are depicted in volcano plots in three pairwise comparisons. (A) *rd6/rd6* (*Mfrp^rd6^*/*Mfrp^rd6^*) P0 vs B6 (C57BL/6J) P0, (B) *rd6/rd6* P14 vs B6 P14 and (C) *rd6/rd6* P14 vs *rd6/rd6* P0.

Gene expression analysis was performed using the Affymetrix Mouse 430v2 microarray. Volcano plots were generated to graphically represent differentially accumulated gene transcripts at a significance level of *q*<0.05 using six pairwise analyses. Comparison of *Mfrp^rd6^* and C57BL/6J samples at the P0 time point did not yield any significant differences in transcript levels, indicating that the effects of *Mfrp* mutation occur postnatally (Fig. 1A). In contrast, comparison at the P14 time point resulted in 2,454 differentially expressed probe sets (Fig. 1B). Not surprisingly, comparison of the P0 and P14 time points in *Mfrp^rd6^* mice yielded 28,452 significant differentially accumulated probe sets (Fig. 1C), and a similar result was obtained in C57BL/6J mice (Fig. S1A). Further analysis of differential accumulated gene transcripts between the two strains (*Mfrp^rd6^* and C57BL/6J) irrespective of the time point yielded a relatively small set of significantly accumulated probe sets (Fig. S1B). By contrast, the comparison of the difference in time point (P0 vs P14) irrespective of the strain difference yielded a relatively large set of significant differentially accumulated probe sets (Fig. S1C). Taken together, this analysis suggests that these probe sets likely change as a consequence of ocular development between P0 and P14.

We generated Venn diagram by comparing all the three sets of data, B6 P14 vs B6 P0, *rd6*/*rd6* P14 vs *rd6*/*rd6* P0 and *rd6*/*rd6* P0 vs *rd6*/*rd6* P14 to determine overlapping and unique genes in each set of comparison (Fig. S2). In *rd6*/*rd6* P14 vs B6 P14 comparison, there were 108 unique set of genes (Fig. S2). When we compared all the three groups, there were 1709 overlapping genes (Fig. S2). B6 P14 vs B6 P0 and *rd6*/*rd6* P14 vs B6 P0 yielded 260 overlapping genes (Fig. S2). Comparison of *rd6*/*rd6* P14 vs *rd6*/*rd6* P0 and *rd6*/*rd6* P0 yielded 377 overlapping genes (Fig. S2).

### Differentially accumulated probe sets in *Mfrp^rd6^* mutant mice at P14

We further analyzed the differentially accumulated probe sets at P14 in *Mfrp^rd6^* mutants relative to controls. Heatmaps were generated for both up- and down-regulated probe sets in *Mfrp^rd6^* mutants. The heatmap color scale corresponds to the fluorescence (log-2, normalized) intensity level of the probe sets, where light blue represents a low level of hybridization to the probe set and dark blue a high level. Upregulated probe sets (5) with a relative fold change >5.0 are listed (Figure S3A). Heatmaps of the down-regulated probe sets at relative fold changes −2.0 to −5.0 include 17 probe sets (Fig. S3B). Heatmaps of downregulated genes at relative fold change −1.5 to −2.0 include 118 probe sets (Fig. S3C).

### Ingenuity pathway analysis

**Figure 2:**
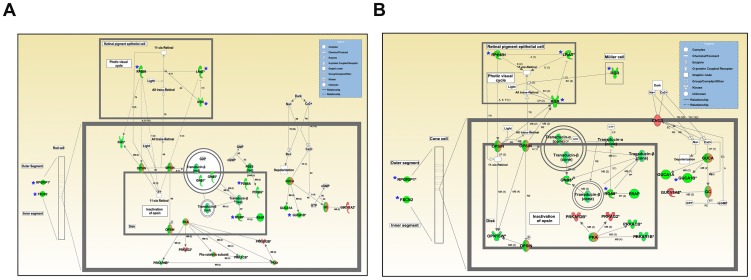
Ingenuity pathway analysis identified Visual Cycle and Phototransduction pathways to be downregulated genes in homozygous *Mfrp^rd6^* mutant mice. (A) Genes in the visual cycle (RPE) and phototransduction pathway (rod photoreceptors) are represented in this panel. The asterisk represents the visual cycle genes (*Rpe65, Lrat and Rgr*), phototransduction pathway genes (*Rgs9*, *Guca1b*, *Pde6a*) and genes encoding structural components of the rod-cells (*Rpgrip1* and *Fscn2*) that were validated by qRT-PCR. (B) Genes in the visual cycle (RPE) and phototransduction pathway (cone photoreceptors) are represented in this panel. The asterisk represents the visual cycle genes (*Rpe65*, *Lrat* and *Rgr*), Müller glia cell expressed gene (*Rgr*), phototransduction pathway genes (*Rgs9* and *Guca1b*), and genes encoding structural components of the cone cells (*RpGrip1* and *Fscn2*) that were validated by qRT-PCR. The molecules associated with the symbols are as depicted in the inset. The solid and dashed lines represent direct or indirect interactions, respectively, between the genes. The arrow indicates interaction between genes. A =  Activation, B = Binding, E = Expression (includes metabolism/synthesis for chemicals), I (Inhibition), PP (Protein-Protein binding), P (Phosphorylation/Dephosphorylation), RB (Regulation of binding), MB (Group/complex Membership).

To determine the molecular networks and biological pathways affected in *Mfrp^rd6^* mutant eye at the P14 time point, we examined the microarray data by Ingenuity Pathway Analysis (IPA). The top five canonical IPA pathways that were altered significantly in *Mfrp^rd6^* mutant eyes were B cell development, allograft rejection signaling, autoimmune thyroid disease signaling, phototransduction, cytotoxic T lymphocyte-mediated apoptosis of target cells, and visual cycle. The genes identified in these pathways and results of statistical tests are given in Table S1. Of particular interest are the phototransduction and visual cycle genes that were perturbed in *Mfrp^rd6^* mutant eyes. The *Mfrp^rd6^* allele, which is a loss of function mutation leading to the absence of MFRP protein from the RPE [8], is likely to have a direct effect on RPE cell function/maintenance. In accordance with this effect, visual cycle gene transcripts expressed in the RPE, including *Rpe65* and *Lrat*, were decreased significantly in *Mfrp^rd6^* mice (Fig. 2, A, B). Transcripts of *Rgr*, which encode a visual cycle protein found in both RPE and Müller cells, were also significantly decreased (Fig. 2B). The *Mfrp^rd6^* mutation also affected retina-specific transcript levels, as evidenced by a significant relative fold change (RFC) of −1.2 to −2.0 in transcripts expressed in photoreceptor cells (Fig. 2, A, B). These included transcripts from genes specifically expressed in rod cells (*Rho*, *Gnb1* and *Gnb5*; Fig. 2A), cone cells (*Opn1sw*, *Gnat2, Gnb3* and *Gnb5*; Fig. 2B), or in both rods and cones (*Rgs9*, *Rgs9bp*, *Prkaca*, *Pde6a, Pde6b, Guca1a and Guca1b*; Fig. 2, A, B). Transcripts encoded by genes implicated in maintaining photoreceptor OS morphology, *Fscn2* and *Rpgrip1*, were also significantly decreased in the *Mfrp^rd6^* mutant (Fig. 2, A, B), consistent with the early OS disorganization that is observed in this mutant.

Selective analysis of our microarray data, examining 250 genes listed in the IPA retinal degeneration (RD) pathway, identified additional transcripts that were differentially regulated in *Mfrp^rd6^* mutants compared to B6 controls at P14. Forty RD pathway gene transcripts were significantly decreased in *Mfrp^rd6^* eyes, with an RFC from −1.15 to −2.39 (Table S2). These include the *C1qtnf5* and *Mertk* transcripts, which are known to be decreased in *Mfrp^rd6^* mice [12]. A further 9 RD pathway gene transcripts were significantly increased in *Mfrp^rd6^* eyes (Table S3), while the remaining 201 failed to show significant change and therefore were not considered further. As RD pathway genes are typically expressed in the retina or RPE, these results suggest that the *Mfrp^rd6^* mutation broadly decreases retina- and RPE-specific transcripts in the posterior eye.

### Validation of differentially accumulated transcripts by qRT-PCR

**Figure 3:**
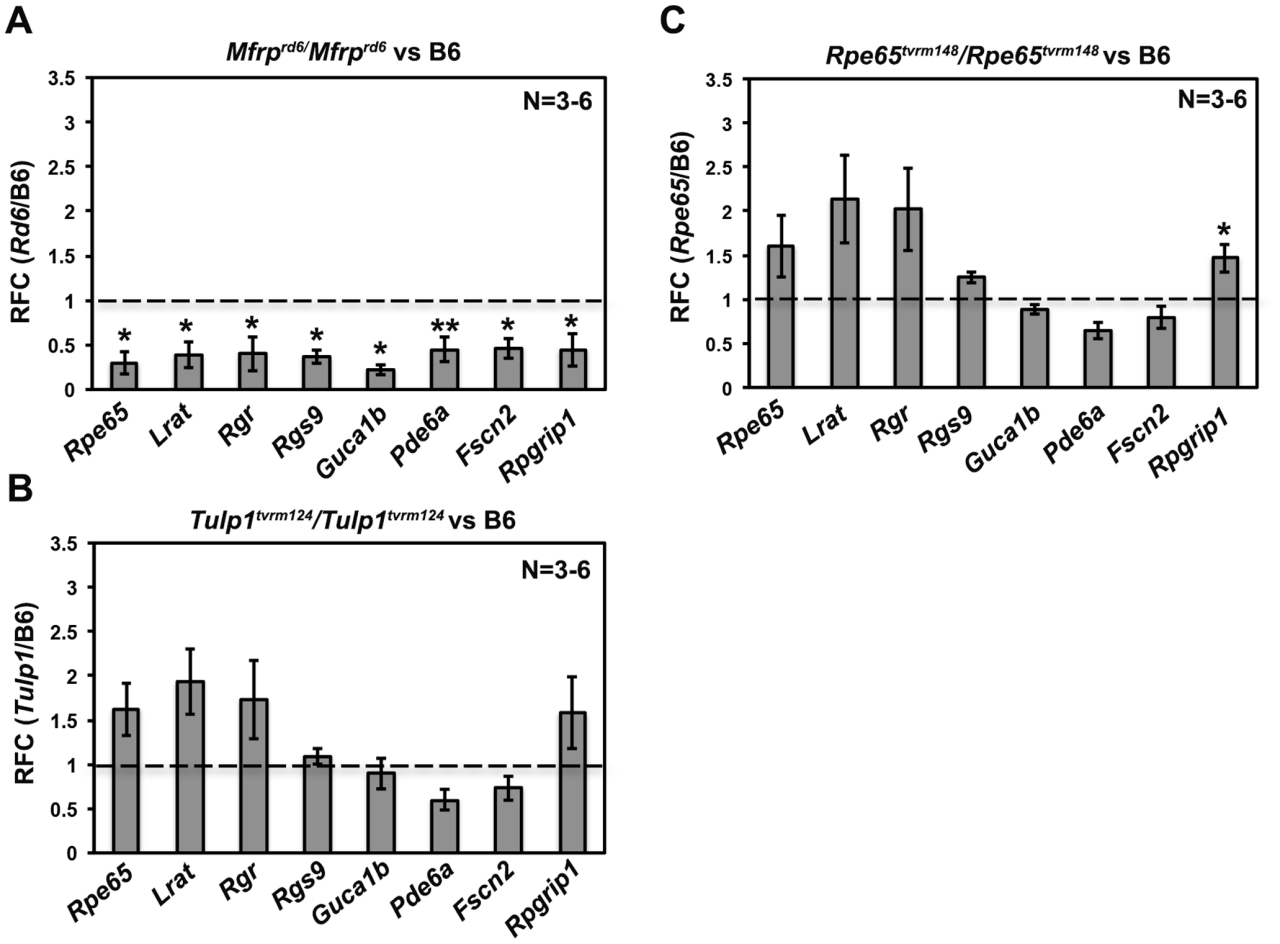
qRT-PCR analysis of RPE and retinal- specific genes in homozygous *Mfrp^rd6^*, *Tulp1^tvrm124^ and Rpe65^tvrm148^* mutants. (A) In homozygous *Mfrp^rd6^* mutant mice, the transcripts in the visual cycle (*Rpe65, Lrat* and *Rgr*), phototransduction pathway (*Rgs9*, *GuCa1b*, *Pde6a*) and structural components of rods and cones (*Fscn2* and *RpGrip1*) were significantly decreased relative to the wild-type control (B6/J), validating the microarray results. (B) qRT-PCR analysis in *Tulp1^tvrm124^/Tulp1^tvrm124^* mutants at P14 revealed no significant change in any of the transcripts tested. (C) In *Rpe65^tvrm148^/Rpe65^tvrm148^* mutants, there was only a significant increase in *RpGrip1* from transcripts tested, relative to wild-type (B6/J) controls. The data are expressed as relative fold change (RFC) after normalizing to the wild-type control. RFC was calculated using ΔΔC_T_ method after internal calibration to β-Actin control. Each value represents RFC ± S.E.M. * *P*<0.05 and ** *P*<0.001 relative to controls. N = 3–6 per group.

To validate the microarray data, qRT-PCR analysis was performed on whole eyes from *Mfrp^rd6^* mutants and C57BL/6J mice. Transcripts that were significantly increased in the microarray analysis are listed in (Table 2). Transcripts that were significantly decreased in the microarray analysis (Table 3), including those encoding components of the visual cycle (*Rpe65*, *Lrat, Rgr*), phototransduction pathway (*Rgs9*, *Pde6a*, *Guca1b*), and involved in disc morphogenesis (*Fscn2*, *Rpgrip1*), were also reduced as determined by qRT-PCR (Fig. 3A). However, some of the changes in transcripts including *Prph2* (−4.1795, *q* value  = 0.06), *Optc* (−2.395, *q*<0.05), *Aqp5* (−2.197, *q*<0.05) (Table 3) were not validated by qRT-PCR. The failure to validate these transcripts was unlikely to be caused by assay limitations. Amplification primers were targeted to exon-exon junctions to amplify only processed RNA (Table 1); a PCR product of the correct size was verified; the PCR efficiency of the primers for genes of interest and calibrator was the same; C_T_ values in control and *Mfrp^rd6^* mutant samples, indicated robust target amplification in both; and melting curve analysis confirmed the presence of a single amplified product. However, the lack of verification may be due the difference in samples used for the microarray and the qRT-PCR analyses, to the low abundance of some transcripts, or in some cases the differences were not significant (e.g. *Prph2*, which is highly expressed in the retina, had a FDR of 0.0605).

**Table 2:** Transcripts that are upregulated in *Mfrp^rd6^* ^/^ *Mfrp^rd6^* mutant relative to B6 control at P14.

Affymetrix Probe Set ID	Gene Symbol	Gene Name	Fold change	FDR	13_Rd6_P14	14_Rd6_P14	15_Rd6_P14	7_B6_P14	8_B6_P14	9_B6_P14	Validated by qRT-PCR	Location	Family
1429647_at	Prss56	protease, serine 56	17.31	0.047	8.87	9.68	10.18	5.64	5.38	5.37	Yes	Cytoplasm	peptidase
1418199_at	Hemgn	hemogen	7.21	0.047	6.3	8.05	6.45	3.98	3.86	4.41	No	Nuclear	other
1425324_x_at	Igh-6	Immunoglobulin heavy chain 6	5.37	0.047	7.61	8.56	7.37	5.32	5.11	5.83	No	Membrane/Secreted	other
1449077_at	Ahsp	alpha hemoglobin stabilizing protein	4.79	0.047	9.26	10.42	8.9	6.76	7.2	7.84	No	Cytoplasm	other
1442459_at	Adamts19	a disintegrin-like and metallopeptidase (reprolysin type) with thrombospondin type 1 motif, 19	4.59	0.047	5.91	6.17	6.97	4.18	4.16	4.11	Yes	Extracellular matrix	other
1418989_at	Ctse	Cathepsin E	3.79	0.047	7.71	7.79	7.2	5.27	5.35	5.97	No	Endosome	other
1417714_x_at	Hba-a1/a2	Hemoglobin alpha adult chain 1/chain 2	3.22	0.047	12.56	13.37	12.41	10.63	11.32	11.33	No	Cytoplasm	other
1419014_at	Rhag	Rhesus blood group-associated A glycoprotein	3.16	0.047	4.93	5.87	4.73	3.09	3.47	3.99	No	Membrane	other
1451675_a_at	Alas2	aminolevulinic acid synthase 2, erythroid	3.06	0.047	8.47	8.87	7.76	6.62	7.26	6.37	No	Mitochondria matrix	other
1416193_at	Car1	carbonic anhydrase 1	2.9	0.047	5.77	7.18	6.1	4.61	4.72	5.11	No	Cytoplasm	enzyme
1423016_a_at	Gypa	glycophorin A	2.57	0.047	4.92	5.83	4.03	3.31	3.62	3.77	No	Membrane	other

The number in the column heading (Table 2) represents the mouse identity used in the microarray analysis.

**Table 3:** Transcripts that are downregulated in *Mfrp^rd6^* ^/^ *Mfrp^rd6^* mutant relative to B6 control at P14.

Affymetrix Probe Set ID	Gene Symbol	Gene Name	Fold change	FDR	13_Rd6_P14	14_Rd6_P14	15_Rd6_P14	7_B6_P14	8_B6_P14	9_B6_P14	Validated by qRT-PCR	Location	Family
1420511_at	*Prph2*	Peripherin 2	−4.18	0.0605	6.32	6.37	11.56	12.05	6.57	11.82	No	Plasma Membrane	transmembrane receptor
1420578_at	*Optc*	Opticin	−2.40	0.0407	7.02	6.78	7.33	8.24	8.29	8.38	No	Extracellular Space	other
1418818_at	*Aqp5*	Aquaporin 5	−2.2	0.0407	9	8.88	8.84	9	8.88	8.84	No	Extracellular Space	transporter
1421345_at	*Lrat*	lecithin retinol acyltransferase (phosphatidylcholine–retinol O-acyltransferase)	−1.82	0.0407	7.72	7.81	8.16	8.97	8.74	8.58	Yes	Cytoplasm	enzyme
1450197_at	*Rpe65*	retinal pigment epithelium-specific protein 65kDa	−1.81	0.0407	9.80	9.61	9.62	10.56	10.57	10.47	Yes	Cytoplasm	enzyme
1425441_at	*Guca1b*	guanylate cyclase activator 1B (retina)	−1.73	0.0407	8.41	8.36	8.30	9.09	9.14	9.20	Yes	Cytoplasm	other
1422832_at	*Rgr*	retinal G protein coupled receptor	−1.62	0.0407	10.52	10.51	10.50	11.20	11.25	11.17	Yes	Plasma Membrane	G-protein coupled receptor
1450415_at	*Pde6a*	phosphodiesterase 6A, cGMP-specific, rod, alpha	−1.55	0.0407	9.12	9.20	9.20	9.61	10.17	9.64	Yes	Plasma Membrane	enzyme
1431357_a_at	*Rpgrip1*	retinitis pigmentosa GTPase regulator interacting protein 1	−1.54	0.0407	9.01	9.15	9.24	9.77	9.82	9.67	Yes	Extracellular Space	other
1440605_at	*Fscn2*	fascin homolog 2, actin-bundling protein, retinal (Strongylocentrotus purpuratus)	−1.52	0.0418	8.39	8.33	7.86	8.71	8.95	8.74	Yes	Cytoplasm	other
1421061_at	*Guca1a*	guanylate cyclase activator 1A (retina)	−1.43	0.0407	10.54	10.47	10.20	10.98	10.93	10.84	Yes	Cytoplasm	other

The number in the column heading (Table 3) represents the mouse identity used in the microarray analysis.

### Quantitative Real Time PCR analysis of visual and phototransduction genes in *Tulp1* and *Rpe65* mutants

To determine if the decrease in the transcript levels of visual and phototransduction genes in the *Mfrp^rd6^* mutant was a non-specific effect of the disease process that occurs during retinal degeneration, we examined the levels of visual cycle and phototransduction pathway transcripts in two unrelated retinal degeneration models with mutations in the retina-specific gene, *Tulp1* or the RPE-specific gene, *Rpe65*. Homozygous *Tulp1* (*Tulp1^tvrm124^*) mutant mice model early onset retinal degeneration and have characteristically shorter OSs at P14 [21], comparable to those observed in *Mfrp^rd6^* mutants. Homozygous null mutation of *Rpe65* (*Rpe65^tvrm148^*) results in slow retinal degeneration and disorganized OS discs at P14 [22], as observed in *Mfrp^rd6^* mice. These three retinal degeneration models show a similar extent of photoreceptor degeneration at P14 (Fig. S4). We reasoned that if the decrease in visual and photoreceptor transcripts observed *in Mfrp^rd6^* mutants were due to non-specific, secondary effects of OS shortening or disorganization, a similar reduction of visual cycle and phototransduction pathway gene transcripts would also be observed. No significant decrease in either visual cycle or phototransduction pathway transcripts were found in either *Tulp1* (Fig. 3B) or *Rpe65* mutant mice (Fig. 3C), suggesting that the observed effects are specific to *Mfrp* disruption.

### Western blot analysis of RPE65 protein in *Mfrp^rd6^* eyes

**Figure 4:**
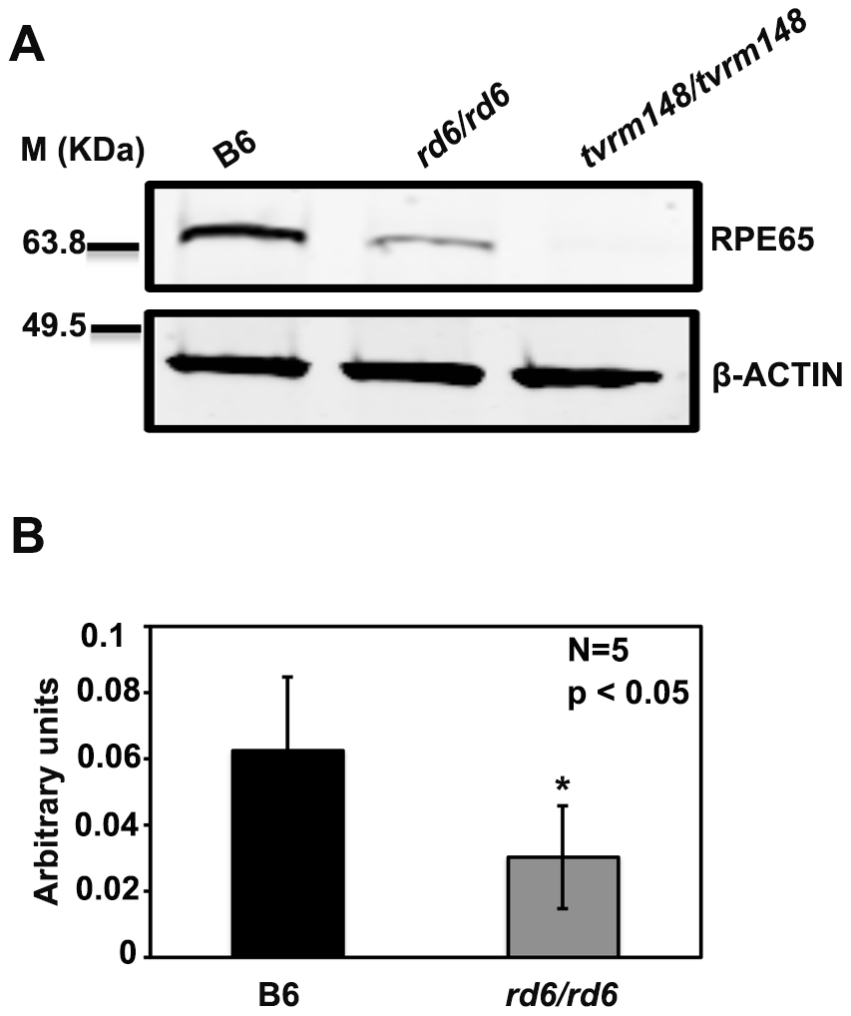
RPE65 protein expression in RPE cells from B6 (C57BL/6J) and homozygous *Mfrp^rd6^* mice. (A) Western blot analysis of RPE65 protein extracted from RPE cells of B6 (C57BL/6J) and homozygous *Mfrp^rd6^* mice. There was a 2.0-fold decrease in RPE65 protein in *Mfrp^rd6^/Mfrp^rd6^* (Lane 2) relative to the B6 control (Lane 1), whereas in *Rpe65^tvrm148^/Rpe65^tvrm148^* mutant, RPE65 protein was undetected (Lane 3). β-Actin loading confirms equal protein loading in all lanes (1–3). (B) Quantitation of RPE65 protein in RPE cells of B6 (C57BL/6J) and *Mfrp^rd6^/Mfrp^rd6^* mice. Student's T test was used to calculate statistical significance (* *P*<0.05 relative to B6 control).

The parallel decrease in retina- and RPE-specific transcripts revealed by transcript analysis raised the possibility that the *Mfrp^rd6^* mutation might diminish retinal health by reducing the levels of RPE visual cycle proteins, which are critical for photoreceptor maintenance [23]. To test this possibility, we examined levels of the *Rpe65* gene product RPE65, a 65 kDa RPE-specific isomerohydrolase that is essential for producing 11-*cis* retinal from all-*trans*-retinyl esters in the visual cycle [23], [24]. Western blot analysis of RPE/choroid/sclera lysates revealed decreased levels of RPE65 protein in *Mfrp^rd6^* mice compared to C57BL/6J controls (Fig. 4A). An *Rpe65* mutant (*Rpe65^tvrm124^/Rpe65^tvrm124^*) was used to control for antibody specificity. No RPE65 protein was detected in lysates from the *Rpe65* mutant mice, whereas a 65 kDa protein was detected in all other samples (Fig. 4A), thus confirming that the detected band is RPE65. Quantitation of the blot indicated a significant 2.0-fold decrease of RPE65 in the *Mfrp^rd6^* mutant eyes (Fig. 4B).

### Increased *Prss56* transcript levels in *Mfrp^rd6^* eyes

Microarray analysis revealed a number of transcripts that accumulated at higher levels in *Mfrp^rd6^* mutant mice compared to wild-type controls. The highest change (17-fold) was observed in *Prss56*, a gene encoding a serine protease (Table 2). Other transcripts involved in hematological (*Hemgn*, *Ahsp*, *Alas2*, *Hba-a1/2*, *Gypa*, *Rhag* and *Car1*) and immune (*Ctse* and *Ighm*) function, were also upregulated (Table 1). However, despite being of sufficient abundance for detection by qRT-PCR, significant differences between wild type and *Mfrp^rd6^* mutants in the latter transcripts were not validated upon further testing.

**Figure 5:**
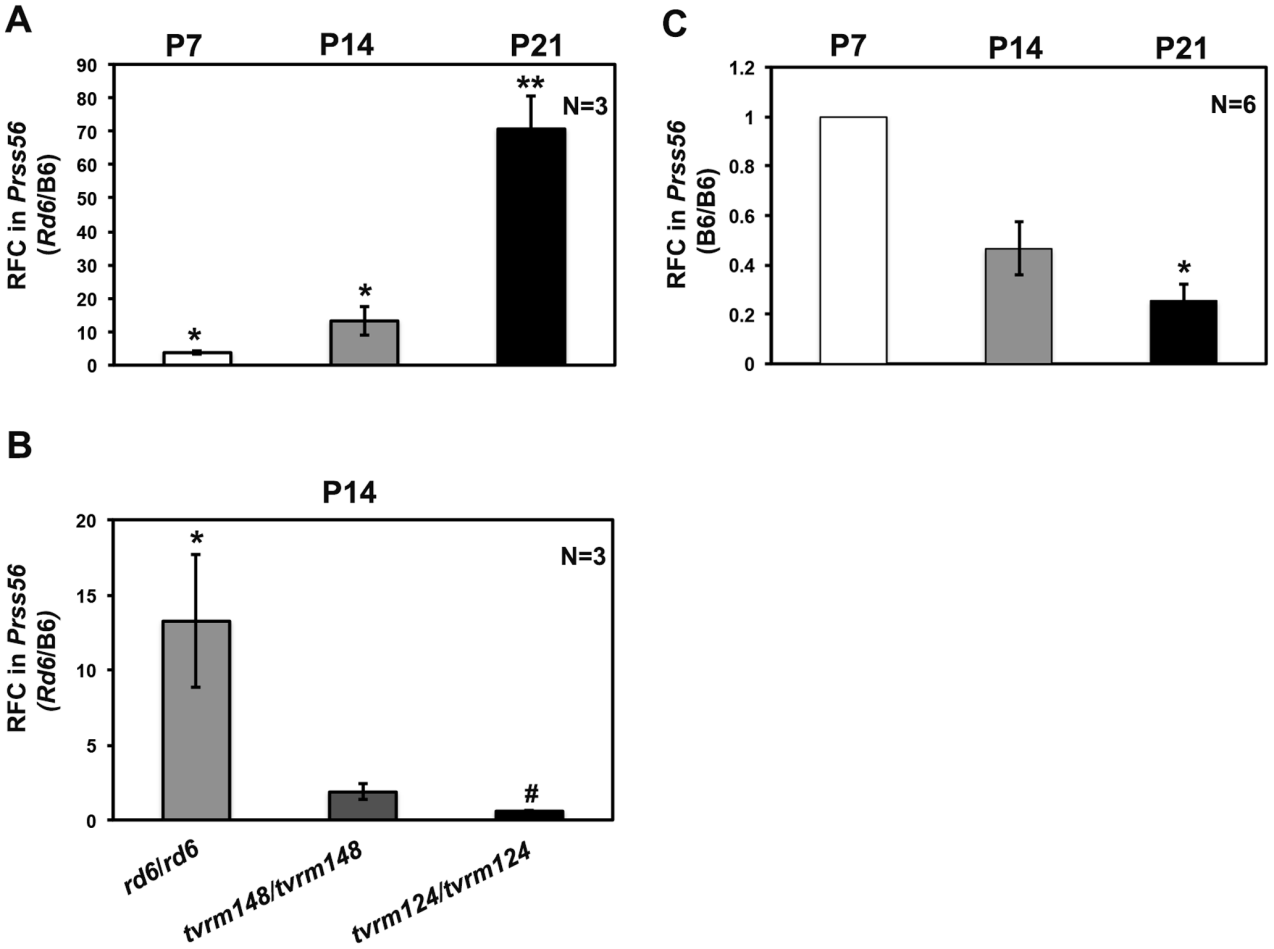
Independent qRT-PCR validation of genes that were differentially expressed in *Mfrp^rd6^/Mfrp^rd6^* mutants by array analysis. The upregulated gene *Prss56* was evaluated at three different time points. (A) At P7, there was a 3.5-fold increase in *Prss56* transcript and it increased to 14-fold by P14, followed by a further 70-fold increase in *Prss56* transcript in *Mfrp^rd6^/Mfrp^rd6^* mutants at P21. (B) At P14, when compared to the *Mfrp^rd6^/Mfrp^rd6^* mutant, in *Tulp1^tvrm124^/Tulp1^tvrm124^* mutants, there was no significant change in *Prss56* transcript, whereas in *Rpe65^tvrm148^/Rpe65^tvrm148^* mutants, there was a significant decrease. (C) Wildtype levels of *Prss56* transcript revealed a significant decrease between P7 and P21 timpoints, whereas the decrease from P7 to P14 was not statistically significant. Data are expressed as relative fold change (RFC) in the *Prss56* transcript after normalizing to the wild-type control (B6). RFC was calculated by the ΔΔC_T_ method using β-actin as an internal calibrator. Each value represents RFC ± S.E.M. * *P*<0.05 and ** *P*<0.001 relative to controls. N = 3 per group.

*PRSS56/Prss56* variants in human and mouse are associated with defects in ocular growth [17]–[19], [25], a process that is also affected by human *MFRP* mutations [1]–[6]. Therefore, we focused on characterizing *Prss56* expression in greater detail. Microarray data indicated no difference in *Prss56* transcript accumulation between *Mfrp^rd6^* mice and controls at P0, suggesting that the increase in *Prss56* expression occurs during postnatal development of the *Mfrp^rd6^* eye. To assess the temporal variation of *Prss56* expression in the postnatal period, qRT-PCR was performed at three different time points (Fig. 5A). At P7, there was a 3.5-fold increase in *Prss56* transcript, which increased to 14-fold at P14 and 70-fold at P21 (Fig. 5A). Thus, the *Mfrp^rd6^* mutation causes a progressive accumulation of *Prss56* transcript throughout postnatal development.

To test whether the increase in *Prss56* expression was a specific attribute of *Mfrp^rd6^* mutant mice, we also examined *Prss56* transcript levels by qRT-PCR in wild type and the *Tulp1* and *Rpe65* mutants. In P14 *Tulp1* mutant mice, there was no significant change in *Prss56* transcript (Fig. 5B), whereas in the *Rpe65* mutant, there was a significant decrease (Fig. 5B). These results suggest that *Prss56* upregulation is specific to homozygous *Mfrp^rd6^* mice. Lastly, we also determined the wildtype level of *Prss56* at P7, P14 and P21 (Fig. 5C). The wildtype level of *Prss56* decreased from P7 to P21 (Fig. 5C). While there was a significant decrease in *Prss56* transcript from P7 to P21, the difference from P7 to P14 was not statistically different (Fig. 5C).

### Cellular localization of *Prss56* and *Glul* in *Mfrp^rd6^*

**Figure 6:**
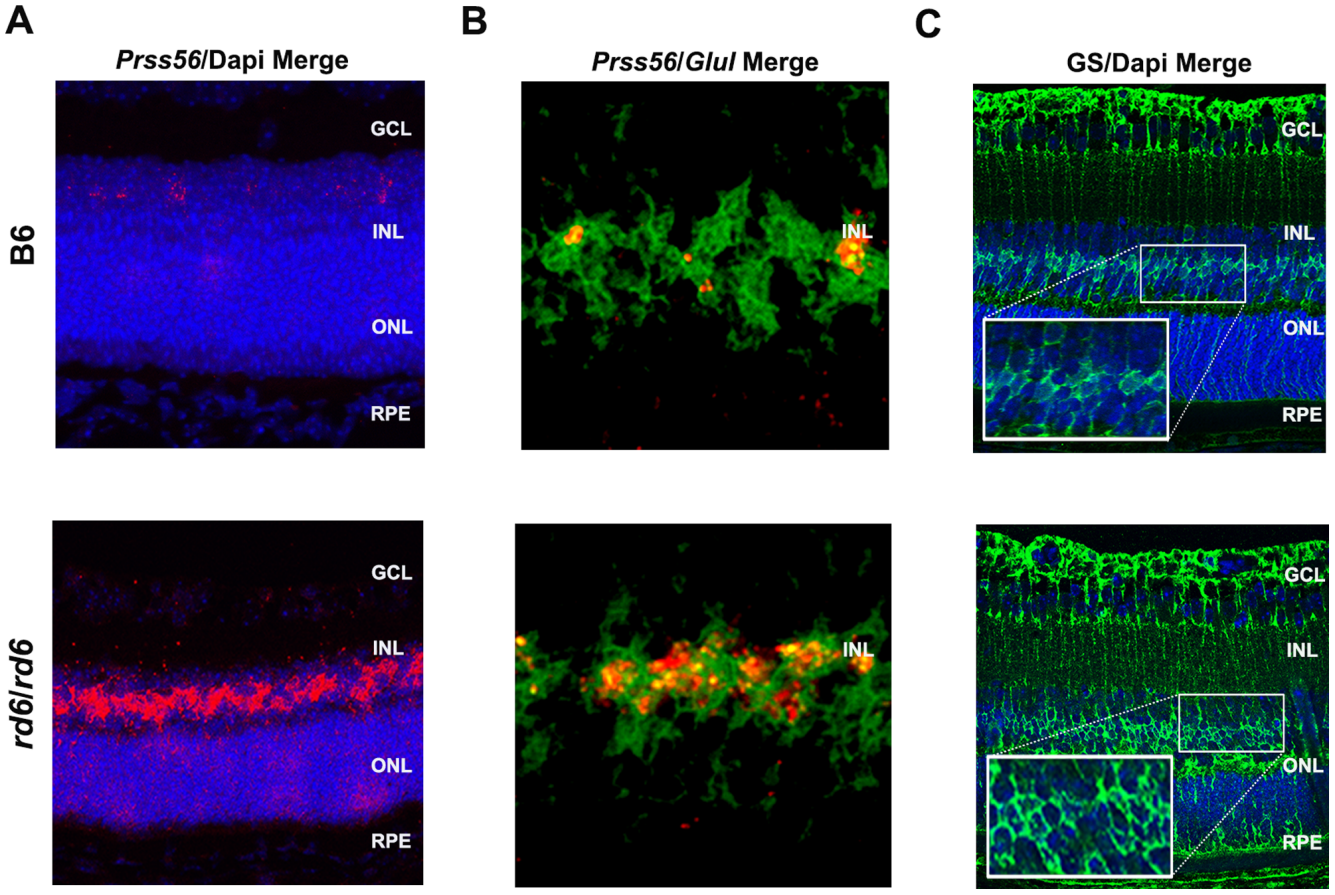
Cellular localization of *Prss56 and Glul* in B6 (C57BL/6J) and *Mfrp^rd6^/Mfrp^rd6^* mice. (A) By *in situ* hybridization, in B6 (C57BL/6J) controls at P14, we observed *Prss56* transcript in only very few cells of the inner nuclear layer (INL) of the retina (top panel), whereas in *Mfrp^rd6^/Mfrp^rd6^* mutants, an intense staining of *Prss56* transcript was observed in INL of the retina (bottom panel). (B) By 2-plex *in situ* hybridization, in B6 controls, we observed co-localization of *Prss56* (red) and *Glul* (pseudo colored green) transcripts in only a few cell body of the (INL) of the retina (top panel), whereas in *Mfrp^rd6^/Mfrp^rd6^*, strong co-localization of *Prss56* and *Glul* transcripts in the cell body of the INL of the retina was observed (bottom panel). (C) Glutamine synthetase (GS) staining of Müller cells. In both C57BL/6J (B6) and *Mfrp^rd6^/Mfrp^rd6^* mice, Müller cells marked with glutamine synthetase showed a similar localization pattern (inset, top and bottom panels) as observed for *Prss56 in situ* hybridization staining, suggesting that Müller cells in the INL of retina express *Prss56* transcripts at P14.

In the absence of an antibody that could reliably detect murine PRSS56, *in situ* hybridization was used to determine the cellular localization of the *Prss56* transcript. In the no probe control, no *Prss56* transcript was observed (data not shown). In the wild-type control at P14, confocal microscopy revealed a few cells in the retinal inner nuclear layer (INL) that showed expression of *Prss56* transcript (Fig. 6A, upper panel). By contrast, in *Mfrp^rd6^* eyes at P14, intense specific staining of the transcript was observed in the INL (Fig. 6A, lower panel). This increased staining further validates both the microarray and qRT-PCR results of increased *Prss56* transcripts in *Mfrp^rd6^* mutant eyes relative to controls.

Further we performed 2-plex *in situ* hybridization using both *Prss56* type 1 probe and *Glul* type 6 probe on the same eye sections. The co-localization of the *Prss56* probe to the same cell bodies expressing *Glul* within the INL of retina in wildtype control (Fig. 6B, upper panel) and *Mfrp^rd6^* (Fig. 6B, lower panel) directly identified Müller cells as specifically expressing *Prss56*.

We also stained eye sections with an antibody to glutamine synthetase, which specifically marks Müller glial cells in the INL. Comparable positive antibody staining of Müller cells was observed in the retinas of both wild type and *Mfrp^rd6^* mice, with a cell body staining pattern in the INL very similar to that observed by *in situ* hybridization of *Prss56* transcripts (Fig. 6C, upper and lower panels). Taken together, these results suggest that Müller cells in the INL express the *Prss56* transcript and are primarily responsible for the observed upregulation of the *Prss56* gene in *Mfrp^rd6^* mice.

## Discussion

Mouse models of retinal degeneration, in which the causative gene has been identified, are important tools for translational vision research [21], as they allow for in-depth study of cellular and molecular changes during development and disease progression. Such studies are especially important when the underlying function of the disrupted protein and molecular basis of the disease or pathology is unknown. Mutations in human *MFRP* [1]–[6] and mouse *Mfrp* [7]–[9] lead to retinal degeneration in both species and have been associated with a decrease in axial length in humans. Although nanophthalmia in *Mfrp* mutants has not been observed, posterior microphthalmia has yet to be assessed. The localization of MFRP to the RPE cell and ciliary body, suggests a potential role in posterior eye homeostasis, however, its function is unclear, and the molecular mechanisms by which mutations of this protein cause disease pathology are unknown. The microarray and validation studies of ocular transcripts in the mouse *Mfrp^rd6^* mutant described in the present study provide new insights into MFRP function.

### Reduced accumulation of retina- and RPE-specific gene transcripts

Microarray analysis identified a modest but significant postnatal decrease in a large number of retina- and RPE-specific gene transcripts in P14 *Mfrp^rd6^* mutant eyes, prior to the degenerative decline in ONL thickness that is associated with photoreceptor cell loss [12]. This result indicates an effect of the mutation beyond the RPE where *Mfrp* is expressed. In our study, the decreased accumulation of posterior eye transcripts was a distinct feature of *Mfrp^rd6^* mice, as qRT-PCR analysis of the *Tulp1* [26] and *Rpe65* [21] mutant models at P14 revealed no significant change in selected visual cycle and phototransduction pathway transcripts that were decreased in *Mfrp^rd6^* mutants. Importantly, our *Tulp1* and *Rpe65* mutant models exhibit a similar time course of retinal degeneration as *Mfrp^rd6^* and show similar OS shortening and disorganization at P14 without a change in ONL thickness. Decreased accumulation of phototransduction pathway transcripts has been documented in other retinal degeneration models [27]–[29]. However, these studies may not be directly comparable to ours, since the disease models were analyzed at ages when ONL thickness was significantly decreased [27], [28] or photoreceptor outer segments were absent [29]. Gene profiling of additional models with a disease progression closely similar to that of *Mfrp^rd6^* may be required to assess whether the decreased accumulation of visual cycle and phototransduction pathway transcripts is a truly distinguishing characteristic of the *Mfrp^rd6^* mutation. Nevertheless, the microarray and qRT-PCR data on *Mfrp^rd6^* and other retinal degeneration mutants suggest a broad and possibly unique role for MFRP protein in postnatal development of the RPE and retina.

A potential explanation for the widespread but modest reduction of visual cycle and phototransduction transcripts in *Mfrp^rd6^* mutants is a defect in the postnatal development of the retina and RPE. The CUB domains, found in MFRP, are prevalent in genes that are developmentally regulated [30]. The CRD domain in MFRP has a high homology to the Frizzled family of proteins, which are normally involved in Wnt signaling and are important in RPE development [31], [32]. Finally, human mutations in *MFRP* have been associated with nanophthalmia or posterior microphthalmia with shortening of the posterior segment of the eye [1]–[6], an expected consequence of reduced retinal and RPE development.

Although no one has actually assessed RPE development in *Mfrp* mutants, apical microvilli defects have been reported [12]. It is interesting to note that during normal eye development, there is a specific and strong increase in *Rpe65* transcription that coincides with the extension of RPE microvilli and the increase in the photoreceptor OS length (reviewed in [24]). Thus, the decrease in *Rpe65* transcript observed in *Mfrp^rd6^* mice may contribute to the decrease in OS length and organization [12]. Moreover, in *Mfrp^rd6^* mutants, the significant decrease in transcripts of *Fscn2*, encoding a protein involved in outer segment morphogenesis, may contribute to the failure to elaborate OS and to the disorganization of OS, followed by photoreceptor degeneration similar to that observed in the *Fscn2* haploinsufficient mouse model [33]. In summary, although we do not know currently how MFRP mediates its effects on the visual cycle and phototransduction genes, it is likely that the observed reductions play a role in the pathogenesis of the disease induced by disruptions in *Mfr*p.

### *Mfrp* and *Prss56*, features of a common pathway?

In this study, we have demonstrated significant upregulation of the retina-specific *Prss56* transcript in *Mfrp^rd6^* eyes. Increased expression was observed during postnatal eye development in Müller cells of *Mfrp^rd6^* mutants, but not in other similarly affected retinal degeneration models, suggesting that *Prss56* upregulation is unique to *Mfrp* disruption. *Prss56* encodes a trypsin-like serine protease [34] of unknown function and substrate specificity in the eye. Interestingly, *PRSS56/Prss56* mutations are associated with autosomal recessive posterior microphthalmia in humans and mice [17], [25], [35]. Moreover, two different genome-wide association studies (GWAS) involving multi-ethnic cohorts identified *Prss56* as significantly associated with refractive errors and myopia [18], [19] that relate to a change in axial length. *MFRP* mutations in humans are also associated with posterior microphthalmia characterized by abnormal posterior segment size leading to hyperopia [2] and cause recessive nanophthalmos [36]. Studies on the postnatal progression of refractive error in nanophthalmos patients having mutations in MFRP suggest a role of MFRP protein in embryonic ocular growth and postnatal emmetropization [36]. As both PRSS56 and MFRP variants affect axial length and potentially the process of emmetropization, it is plausible that they may function through a common biological pathway, yet to be determined.

Like other serine proteases [37], PRSS56 may either directly or indirectly be involved in extracellular matrix (ECM) processing, degradation and remodeling, as suggested previously [17]. Accordingly, upregulation of *Prss56* expression in *Mfrp^rd6^* mutants may promote ECM remodeling. Matrix metalloproteinases are also thought to play an important role in eye development and disease [38]. Consistent with enhanced metalloproteinase activity, IPA analysis of microarray data revealed a significant decrease in *Timp3* transcripts (Table S2), which encode tissue inhibitor of metalloproteinase-3, and a significant increase in *Adamts19* (Table 1) transcripts, which encode a disintegrin and metalloproteinase with thrombospondin motif family member. Taken together, these findings suggest that altered ECM processing may contribute to the progressive loss of photoreceptor cells in the *Mfrp^rd6^* mutant, as observed in other mouse retinal degeneration models [39].

In conclusion, the present study suggests a broad role of MFRP in determining retinal and RPE transcript levels during postnatal development. Most importantly, the upregulation of *Prss56* expression in *Mfrp^rd6^* Müller cells suggests a possible interaction between *Mfrp* and *Prss56* in posterior eye maintenance and development during this period. Future studies would be directed toward understanding how MFRP influences transcript accumulation in the postnatal RPE and retina, and also address how MFRP and PRSS56 interact.

## Supporting Information

Figure S1 **Volcano plots showing the relationship between fold change (represented as mean A-mean B) and the level of significance (represented by the Fs permutated p-value).** Differentially expressed probe sets (q<0.05 shown in red across all fold change levels) at and fold change greater than 2 are depicted in volcano plots in three pairwise comparisons. (A) B6 (C57BL/6J) P14 vs B6 (C57BL/6J) P0, (B) B6 (C57BL/6J) P14; *rd6/rd6* (*Mfrp^rd6^*) P14 vs B6 (C57BL/6J) P0; *rd6/rd6* (*Mfrp^rd6^*)P0 and (C) *rd6/rd6* (*Mfrp^rd6^*) P0; *rd6/rd6* (*Mfrp^rd6^*) P14 vs B6 (C57BL/6J) P0; B6 (C57BL/6J) P14.
(TIF)

Figure S2 **Venn diagram depicting the overlapping and unique genes in the three data sets.** *rd6/rd6* (*Mfrp^rd6^*) P14 vs B6 (C57BL/6J) P14; *rd6/rd6* (*Mfrp^rd6^*) P14 vs *rd6/rd6* (*Mfrp^rd6^*)P0 and B6 (C57BL/6J) P14 vs B6 (C57BL/6J) P0.
(TIF)

Figure S3 **Heat maps of differentially expressed genes in** ***Mfrp^rd6/rd6^*** **mice in comparison to age matched WT controls.** (A) Upregulated genes in *rd6/rd6* (*Mfrp^rd6^*) P14 vs B6 (C57BL/6J) P14, RFC>5.0, q<0.05 (B) Downregulated genes in *rd6/rd6* (*Mfrp^rd6^*) P14 vs B6 (C57BL/6J) P14, RFC <−2.0, q<0.05 (C) Downregulated genes in *rd6/rd6* (*Mfrp^rd6^*) P14 vs B6 (C57BL/6J) P14, RFC <−1.5 to −2.0, q<0.05. Asterisk denotes the genes that were validated by qRT-PCR analysis.
(TIF)

Figure S4 **Outer segment degeneration at P14 in** ***Mfrp^rd6^*** ^**/*rd6***^ **,** ***Tulp1^tvrm124^*** ^**/*tvrm124***^ **,** ***Rpe65^tvrm148^*** ^**/t*vrm148***^ **mice compared to age matched controls visualized by light microscopy.** Retinal sections at p14 were obtained from B6 (A), *Tulp1^tvrm124^* ^/*tvrm124*^ (B) *Mfrp^rd6^* ^/*rd6*^ (C) and *Rpe65^tvrm148^* ^/t*vrm148*^ (D) and stained with hematoxylin & eosin. GC, gangalion cell layer; INL, inner nuclear layer; ONL, outer nuclear layer; OS, outer segments. Magnification: 20 x.
(TIF)

Table S1 **Canonical pathways identified in** ***Mfrp^rd6^*** **mice.**
(DOCX)

Table S2 **Transcripts in retinal degeneration pathway that is downregulated in** ***Mfrp^rd6^*** **mutant mice at P14.**
(DOCX)

Table S3 **Transcripts in retinal degeneration pathway that is up-regulated in** ***Mfrp^rd6^*** **mutant mice at P14.**
(DOCX)
