# Supplementary figures and images for: Gene Profiling of Postnatal Mfrprd6 Mutant Eyes Reveals Differential Accumulation of Prss56, Visual Cycle and Phototransduction mRNAs
Source: PLoS One. 2014 Oct 30;9(10):e110299. doi: 10.1371/journal.pone.0110299 (PMC4214712; doi:10.1371/journal.pone.0110299)

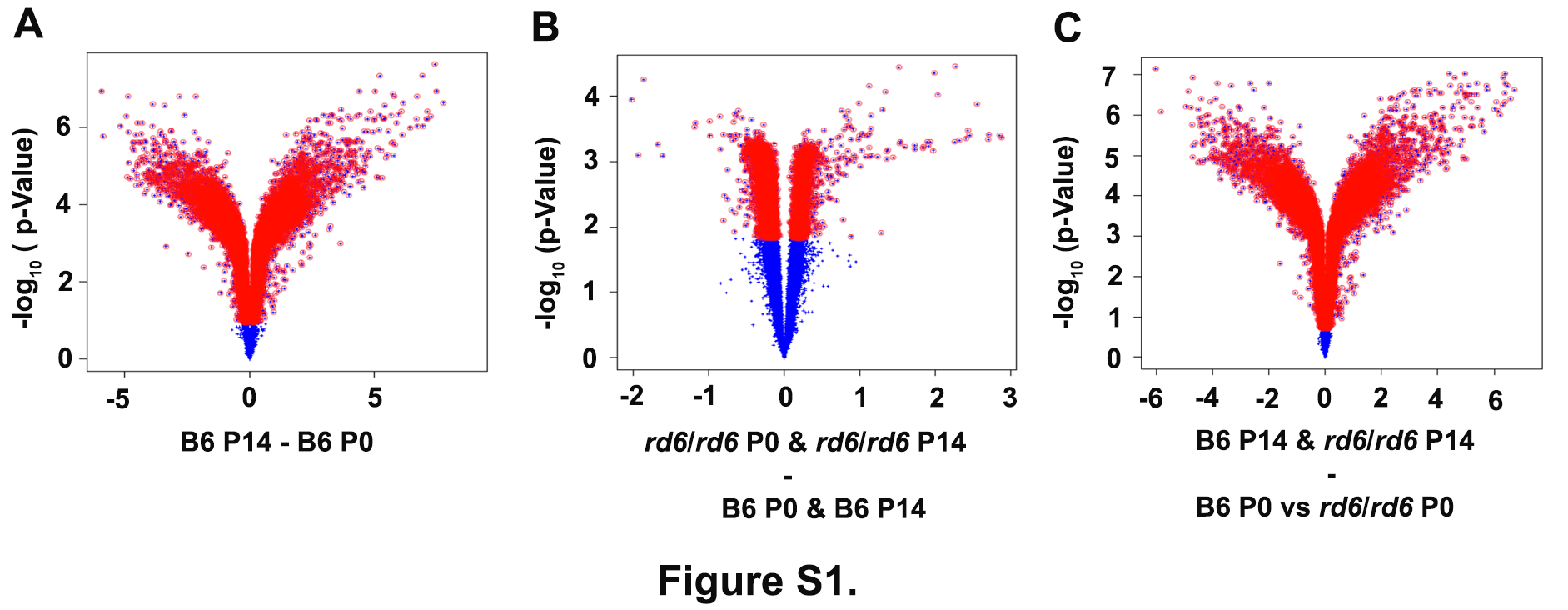

Supplement: Figure S1 — Volcano plots showing the relationship between fold change (represented as mean A-mean B) and the level of significance (represented by the Fs permutated p-value). Differentially expressed probe sets (q<0.05 shown in red across all fold change levels) at and fold change greater than 2 are depicted in volcano plots in three pairwise comparisons. (A) B6 (C57BL/6J) P14 vs B6 (C57BL/6J) P0, (B) B6 (C57BL/6J) P14; rd6/rd6 (Mfrprd6) P14 vs B6 (C57BL/6J) P0; rd6/rd6 (Mfrprd6)P0 and (C) rd6/rd6 (Mfrprd6) P0; rd6/rd6 (Mfrprd6) P14 vs B6 (C57BL/6J) P0; B6 (C57BL/6J) P14. (TIF) [file pone.0110299.s001.tif]

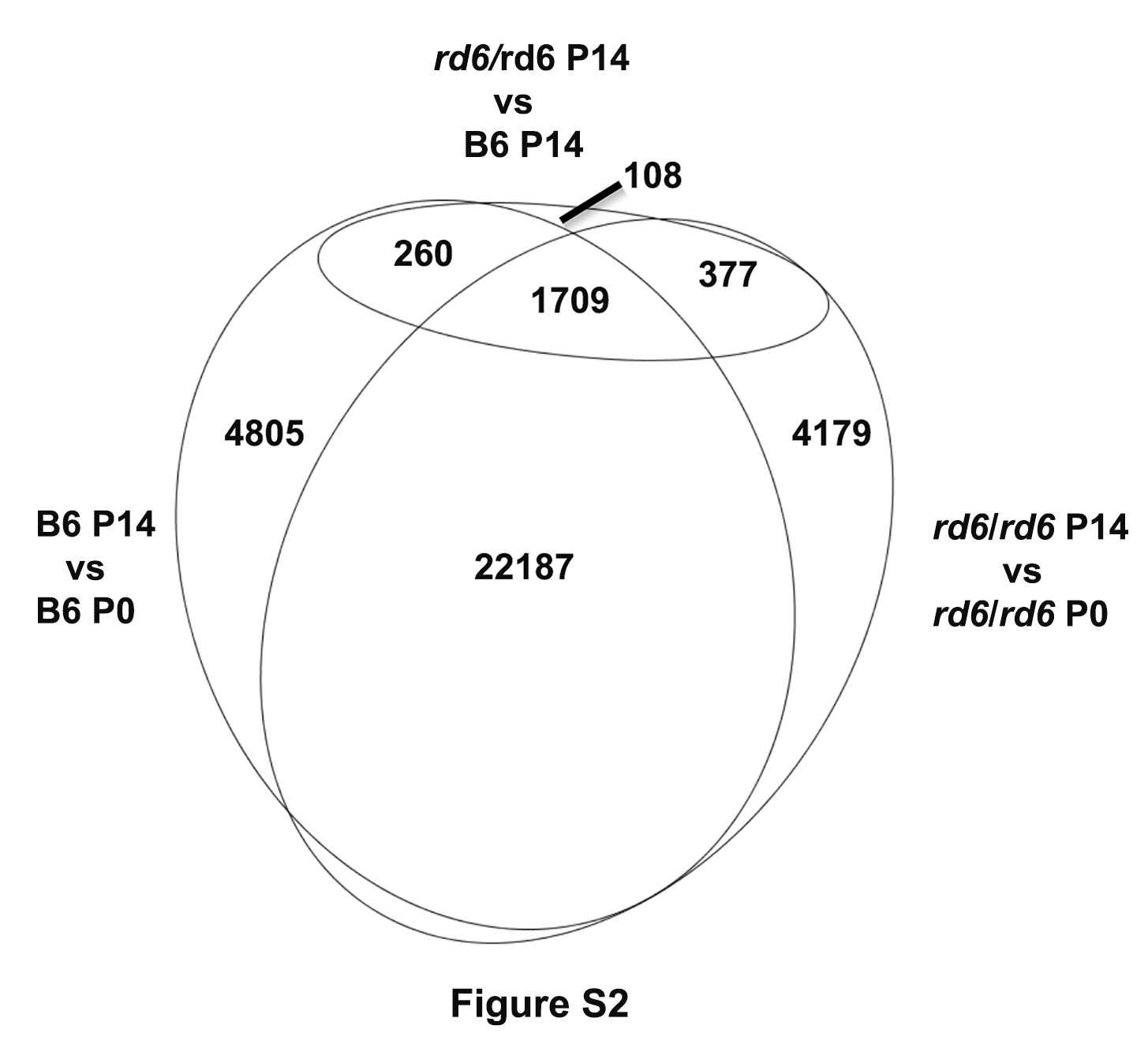

Supplement: Figure S2 — Venn diagram depicting the overlapping and unique genes in the three data sets. rd6/rd6 (Mfrprd6) P14 vs B6 (C57BL/6J) P14; rd6/rd6 (Mfrprd6) P14 vs rd6/rd6 (Mfrprd6)P0 and B6 (C57BL/6J) P14 vs B6 (C57BL/6J) P0. (TIF) [file pone.0110299.s002.tif]

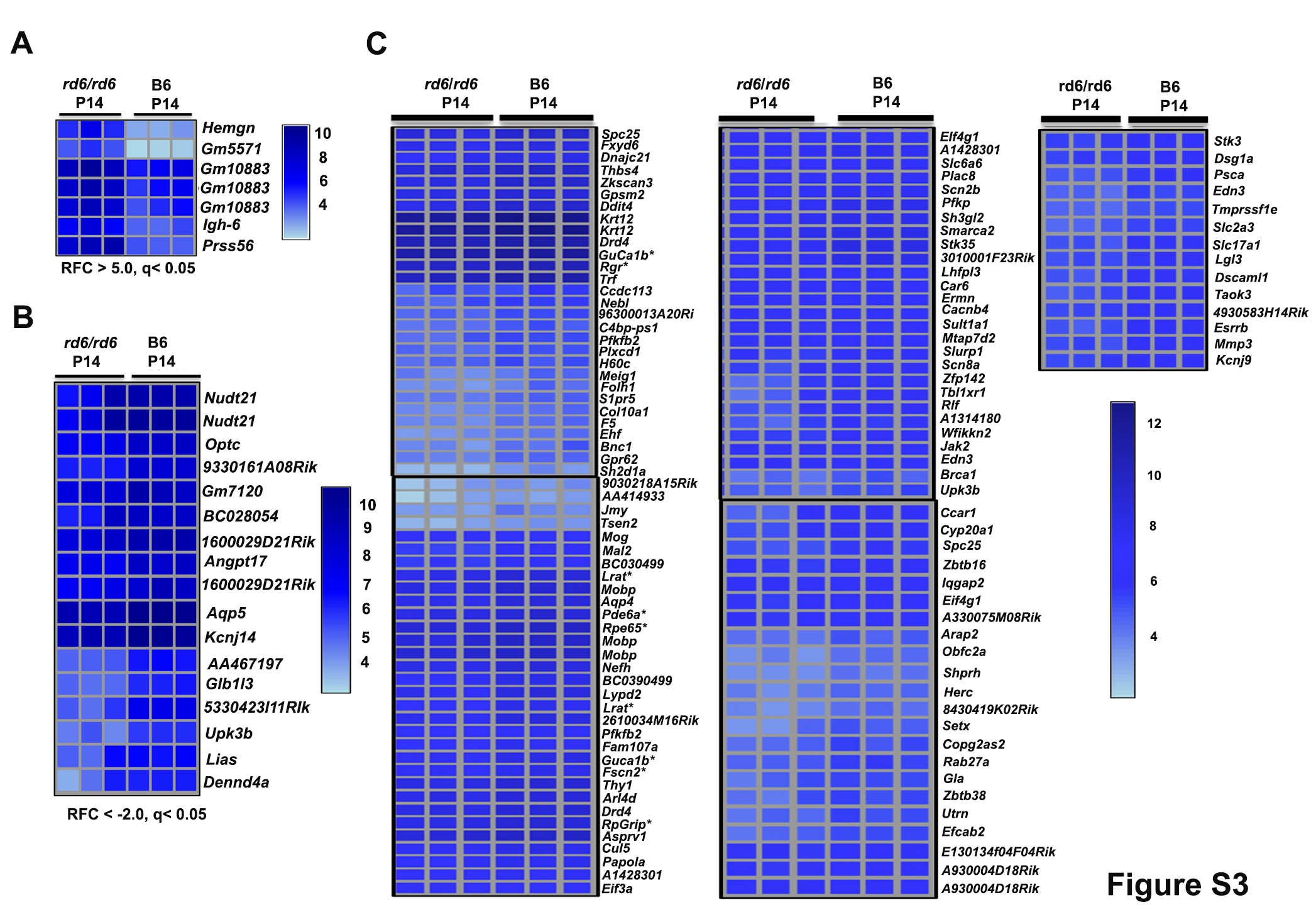

Supplement: Figure S3 — Heat maps of differentially expressed genes in Mfrprd6/rd6 mice in comparison to age matched WT controls. (A) Upregulated genes in rd6/rd6 (Mfrprd6) P14 vs B6 (C57BL/6J) P14, RFC>5.0, q<0.05 (B) Downregulated genes in rd6/rd6 (Mfrprd6) P14 vs B6 (C57BL/6J) P14, RFC <−2.0, q<0.05 (C) Downregulated genes in rd6/rd6 (Mfrprd6) P14 vs B6 (C57BL/6J) P14, RFC <−1.5 to −2.0, q<0.05. Asterisk denotes the genes that were validated by qRT-PCR analysis. (TIF) [file pone.0110299.s003.tif]

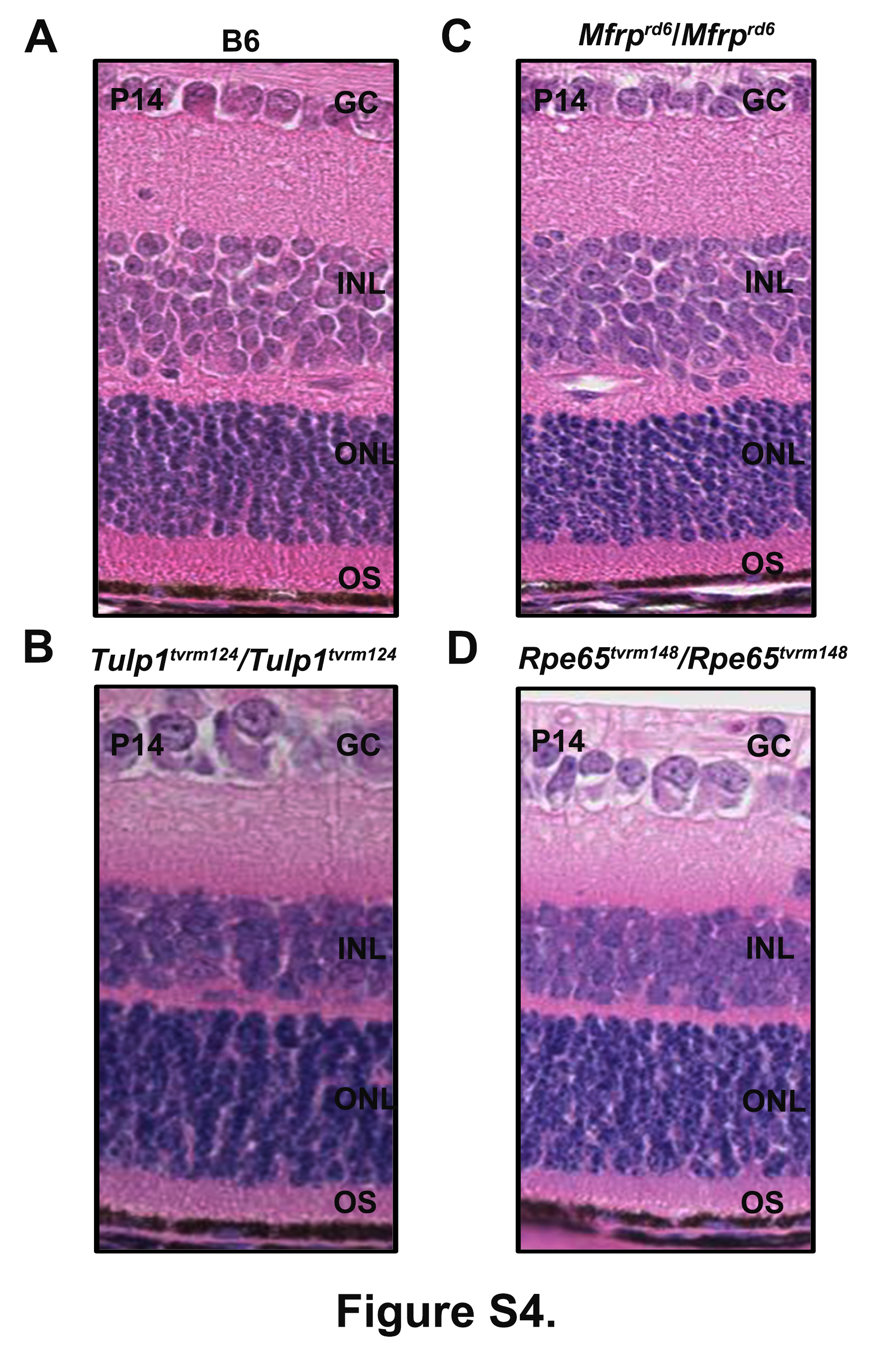

Supplement: Figure S4 — Outer segment degeneration at P14 in Mfrprd6 /rd6 , Tulp1tvrm124 /tvrm124 , Rpe65tvrm148 /tvrm148 mice compared to age matched controls visualized by light microscopy. Retinal sections at p14 were obtained from B6 (A), Tulp1tvrm124 /tvrm124 (B) Mfrprd6 /rd6 (C) and Rpe65tvrm148 /tvrm148 (D) and stained with hematoxylin & eosin. GC, gangalion cell layer; INL, inner nuclear layer; ONL, outer nuclear layer; OS, outer segments. Magnification: 20 x. (TIF) [file pone.0110299.s004.tif]
