# Supplementary material for: Gene Profiling of Postnatal Mfrprd6 Mutant Eyes Reveals Differential Accumulation of Prss56, Visual Cycle and Phototransduction mRNAs
Source: PLoS One. 2014 Oct 30;9(10):e110299. doi: 10.1371/journal.pone.0110299 (PMC4214712; doi:10.1371/journal.pone.0110299)
Supplement: Table S1 — Canonical pathways identified in Mfrprd6 mice. (DOCX) [file pone.0110299.s005.docx]

**Table S1.** Canonical pathways identified in *Mfrp^rd6^* mice

| **Canonical pathways** | **Gene Symbol** | **P-value** | **Ratio** |
| --- | --- | --- | --- |
| B cell development | *Hla-dq1a,* *Hla-dqb1*, *Ighm, Ptprc* . | 6.49E-04 | 4/33  (0.121) |
| Allograft rejection signaling | *Hr-t24*, *Hla-b*, *Hla-dqa1*, *Hladqb1*, I*ghg*1. | 6.76E-04 | 5/95 (0.053) |
| Autoimmune thyroid disease signaling | *Hla-b*, *Hladqa1*, *Hladqb1*, *Ighg1* . | 2.48E-03 | 4/61 (0.066) |
| Phototransduction pathway/  Visual cycle | *Guca1b*, *Pde6a*, *Rgr*, *Rgs9*.  *Rpe65*, *Lrat* . | 6.37E-03  (4.07E-02) | 4/65 (0.062)  2/28 (0.071) |
| Cytotoxic T lymphocyte -mediated apoptosis of target cells | *H2T24*, *Hla-b*, *Hla-dqa1*, *Hla-dqb1* . | 6.9E.03 | 4/85  (0.0471) |
