# Supplementary material for: Gene Profiling of Postnatal Mfrprd6 Mutant Eyes Reveals Differential Accumulation of Prss56, Visual Cycle and Phototransduction mRNAs
Source: PLoS One. 2014 Oct 30;9(10):e110299. doi: 10.1371/journal.pone.0110299 (PMC4214712; doi:10.1371/journal.pone.0110299)
Supplement: Table S2 — Transcripts in retinal degeneration pathway that is downregulated in Mfrprd6 mutant mice at P14. (DOCX) [file pone.0110299.s006.docx]

**Table S2**. Transcripts in retinal degeneration pathway that are downregulated in *Mfrp^rd6^* mutant mice at P14.

| **Affymetrix Probe Set ID** | **Gene Symbol** | **Gene Name** | **Fold change** | **FDR** | **13_Rd6_P14** | **14_Rd6_P14** | **15_Rd6_P14** | **7_B6_P14** | **8_B6_P14** | **9_B6_P14** | **Location** | **Family** |
| --- | --- | --- | --- | --- | --- | --- | --- | --- | --- | --- | --- | --- |
| 1420511_at | *Prph2* | Peripherin 2 | -4.18 | 0.0605 | 6.32 | 6.37 | 11.56 | 12.05 | 6.57 | 11.82 | Plasma Membrane | transmembrane receptor |
| 1420578_at | *Optc* | Opticin | -2.40 | 0.0407 | 7.02 | 6.78 | 7.33 | 8.24 | 8.29 | 8.38 | Extracellular Space | other |
| 1418818_at | *Aqp5* | Aquaporin 5 | -2.2 | 0.0407 | 9 | 8.88 | 8.84 | 9 | 8.88 | 8.84 | Plasma Membrane | ion channel |
| 1425546_a_at | *Trf* | Transferrin | -1.98 | 0.0407 | 10.62 | 10.78 | 10.74 | 11.68 | 11.72 | 11.69 | Extracellular Space | transporter |
| 1422986_at | *Esrrb* | estrogen-related receptor beta | -1.84 | 0.0407 | 6.08 | 5.88 | 6.19 | 6.90 | 7.03 | 6.87 | Nucleus | ligand-dependent nuclear receptor |
| 1421345_at | *Lrat* | lecithin retinol acyltransferase (phosphatidylcholine--retinol O-acyltransferase) | -1.82 | 0.0407 | 7.72 | 7.81 | 8.16 | 8.97 | 8.74 | 8.58 | Cytoplasm | enzyme |
| 1450197_at | *Rpe65* | retinal pigment epithelium-specific protein 65kDa | -1.81 | 0.0407 | 9.80 | 9.61 | 9.62 | 10.56 | 10.57 | 10.47 | Cytoplasm | enzyme |
| 1425441_at | *Guca1b* | guanylate cyclase activator 1B (retina) | -1.73 | 0.0407 | 8.41 | 8.36 | 8.30 | 9.09 | 9.14 | 9.20 | Cytoplasm | other |
| 1422832_at | *Rgr* | retinal G protein coupled receptor | -1.62 | 0.0407 | 10.52 | 10.51 | 10.50 | 11.20 | 11.25 | 11.17 | Plasma Membrane | G-protein coupled receptor |
| 1420148_at | *Slc6a6* | solute carrier family 6 (neurotransmitter transporter), member 6 | -1.61 | 0.0407 | 7.57 | 7.33 | 7.71 | 8.06 | 8.40 | 8.22 | Plasma Membrane | transporter |
| 1450415_at | *Pde6a* | phosphodiesterase 6A, cGMP-specific, rod, alpha | -1.55 | 0.0407 | 9.12 | 9.20 | 9.20 | 9.61 | 10.17 | 9.64 | Plasma Membrane | enzyme |
| 1431357_a_at | *Rpgrip1* | retinitis pigmentosa GTPase regulator interacting protein 1 | -1.54 | 0.0407 | 9.01 | 9.15 | 9.24 | 9.77 | 9.82 | 9.67 | Extracellular Space | other |
| 1440605_at | *Fscn2* | fascin homolog 2, actin-bundling protein, retinal (Strongylocentrotus purpuratus) | -1.52 | 0.0418 | 8.39 | 8.33 | 7.86 | 8.71 | 8.95 | 8.74 | Cytoplasm | other |
| 1450041_a_at | *Tub* | tubby bipartite transcription factor | -1.47 | 0.0415 | 6.30 | 6.17 | 6.36 | 6.97 | 7.07 | 6.47 | Cytoplasm | transcription regulator |
| 1459268_at | *Gpr179* | G protein-coupled receptor 179 | -1.46 | 0.0407 | 7.28 | 7.33 | 7.38 | 7.98 | 7.71 | 7.95 | Plasma Membrane | G-protein coupled receptor |
| 1449334_at | *Timp3* | TIMP metallopeptidase inhibitor 3 | -1.44 | 0.0407 | 9.25 | 9.37 | 9.69 | 10.00 | 9.81 | 10.07 | Extracellular Space | other |
| 1421061_at | *Guca1a* | guanylate cyclase activator 1A (retina) | -1.43 | 0.0407 | 10.54 | 10.47 | 10.20 | 10.98 | 10.93 | 10.84 | Cytoplasm | other |
| 1460386_a_at | *Slc1a1* | solute carrier family 1 (neuronal/epithelial high affinity glutamate transporter, system Xag), member 1 | -1.43 | 0.0407 | 7.38 | 7.56 | 7.61 | 7.96 | 8.11 | 8.02 | Plasma Membrane | transporter |
| 1417754_at | *Topors* | topoisomerase I binding, arginine/serine-rich, E3 ubiquitin protein ligase | -1.43 | 0.0407 | 7.06 | 7.22 | 7.51 | 7.83 | 7.71 | 7.79 | Nucleus | enzyme |
| 1456418_at | *Kcnj13* | potassium inwardly-rectifying channel, subfamily J, member 13 | -1.39 | 0.0407 | 9.82 | 9.66 | 9.69 | 10.12 | 10.24 | 10.24 | Plasma Membrane | ion channel |
| 1439083_at | *Ahi1* | Abelson helper integration site 1 | -1.38 | 0.0407 | 7.26 | 7.29 | 7.39 | 7.68 | 7.87 | 7.79 | Cytoplasm | other |
| 1425590_s_at | *Aipl1* | aryl hydrocarbon receptor interacting protein-like 1 | -1.38 | 0.0407 | 11.23 | 11.48 | 11.47 | 11.89 | 11.86 | 11.83 | Nucleus | other |
| 1421085_at | *Rs1* | retinoschisin 1 | -1.36 | 0.0455 | 9.23 | 9.10 | 9.35 | 9.72 | 9.66 | 9.64 | Extracellular Space | other |
| 1460498_a_at | *Dnajc5* | DnaJ (Hsp40) homolog, subfamily C, member 5 | -1.36 | 0.0434 | 7.25 | 7.50 | 7.77 | 7.91 | 7.99 | 7.95 | Plasma Membrane | other |
| 1453385_at | *Xiap* | X-linked inhibitor of apoptosis | -1.36 | 0.0490 | 4.62 | 5.05 | 4.97 | 5.31 | 5.09 | 5.56 | Cytoplasm | enzyme |
| 1425821_at | *Clcn7* | chloride channel, voltage-sensitive 7 | -1.35 | 0.0408 | 5.72 | 5.83 | 6.11 | 6.30 | 6.25 | 6.41 | Plasma Membrane | ion channel |
| 1423239_at | *Impdh1* | IMP (inosine 5'-monophosphate) dehydrogenase 1 | -1.34 | 0.0407 | 10.04 | 10.09 | 10.01 | 10.50 | 10.44 | 10.48 | Cytoplasm | enzyme |
| 1425249_a_at | *Tyro3* | TYRO3 protein tyrosine kinase | -1.32 | 0.0407 | 6.59 | 6.77 | 6.68 | 7.04 | 7.04 | 7.15 | Plasma Membrane | kinase |
| 1453860_s_at | *Nr3c1* | nuclear receptor subfamily 3, group C, member 1 (glucocorticoid receptor) | -1.31 | 0.0407 | 6.31 | 6.51 | 6.54 | 6.89 | 6.73 | 6.92 | Nucleus | ligand-dependent nuclear receptor |
| 1451156_s_at | *Vldlr* | very low density lipoprotein receptor | -1.31 | 0.0407 | 6.30 | 6.22 | 6.47 | 6.78 | 6.72 | 6.65 | Plasma Membrane | transporter |
| 1429859_a_at | *Arl2bp* | ADP-ribosylation factor-like 2 binding protein | -1.24 | 0.0457 | 9.49 | 9.63 | 9.72 | 9.93 | 9.97 | 9.86 | Cytoplasm | transcription regulator |
| 1424478_at | *Bbs2* | Bardet-Biedl syndrome 2 | -1.24 | 0.0407 | 9.15 | 9.21 | 9.11 | 9.48 | 9.53 | 9.38 | Cytoplasm | other |
| 1451617_at | *Rho* | rhodopsin | -1.23 | 0.0407 | 13.13 | 13.10 | 13.18 | 13.45 | 13.41 | 13.46 | Plasma Membrane | G-protein coupled receptor |
| 1458626_at | *Nos1* | nitric oxide synthase 1 (neuronal) | -1.22 | 0.0407 | 4.01 | 4.03 | 4.01 | 4.30 | 4.33 | 4.29 | Cytoplasm | enzyme |
| 1450453_a_at | *Pde6g* | phosphodiesterase 6G, cGMP-specific, rod, gamma | -1.21 | 0.0420 | 12.89 | 13.05 | 12.85 | 13.18 | 13.21 | 13.22 | Cytoplasm | enzyme |
| 1451653_a_at | *Fam161a* | family with sequence similarity 161, member A | -1.21 | 0.0488 | 9.72 | 9.86 | 9.75 | 10.08 | 10.02 | 10.04 | Cytoplasm | other |
| 1453367_a_at | *Abhd12* | abhydrolase domain containing 12 | -1.20 | 0.0407 | 9.37 | 9.38 | 9.43 | 9.67 | 9.73 | 9.58 | Other | enzyme |
| 1422869_at | *Mertk* | c-mer proto-oncogene tyrosine kinase | -1.20 | 0.0407 | 7.51 | 7.52 | 7.41 | 7.77 | 7.78 | 7.67 | Plasma Membrane | kinase |
| 1432466_a_at | *Apoe* | apolipoprotein E | -1.19 | 0.0422 | 12.27 | 12.48 | 12.36 | 12.64 | 12.62 | 12.60 | Extracellular Space | transporter |
| 1424762_at | *C1qtnf5* | C1q and tumor necrosis factor related protein 5 | -1.19 | 0.0418 | 9.22 | 9.24 | 9.15 | 9.43 | 9.47 | 9.46 | Plasma Membrane | transmembrane receptor |
| 1453005_at | *Prpf31* | pre-mRNA processing factor 31 | -1.18 | 0.0407 | 7.66 | 7.57 | 7.68 | 7.88 | 7.87 | 7.87 | Nucleus | other |
| 1451582_at | *Tulp1* | tubby like protein 1 | -1.15 | 0.0420 | 12.12 | 12.20 | 12.21 | 12.38 | 12.42 | 12.33 | Nucleus | transcription regulator |

The number in the column heading (Table S2) represents the mouse identity used in the microarray analysis.
