# Supplementary material for: Gene Profiling of Postnatal Mfrprd6 Mutant Eyes Reveals Differential Accumulation of Prss56, Visual Cycle and Phototransduction mRNAs
Source: PLoS One. 2014 Oct 30;9(10):e110299. doi: 10.1371/journal.pone.0110299 (PMC4214712; doi:10.1371/journal.pone.0110299)
Supplement: Table S3 — Transcripts in retinal degeneration pathway that is up-regulated in Mfrprd6 mutant mice at P14. (DOCX) [file pone.0110299.s007.docx]

**Table S3**. Transcripts in retinal degeneration pathway that are up-regulated in *Mfrp^rd6^* mutant mice at P14.

| **Affymetrix Probe Set ID** | **Gene Symbol** | **Gene Name** | **Fold change** | **FDR** | **13_Rd6_P14** | **14_Rd6_P14** | **15_Rd6_P14** | **7_B6_P14** | **8_B6_P14** | **9_B6_P14** | **Location** | **Family** |
| --- | --- | --- | --- | --- | --- | --- | --- | --- | --- | --- | --- | --- |
| 1460708_s_at | *Cdc42* | cell division cycle 42 | 1.13 | 0.0487 | 11.5 | 11.56 | 11.6 | 11.4 | 11.36 | 11.38 | Cytoplasm | enzyme |
| 1427467_a_at | *Rpgr* | retinitis pigmentosa GTPase regulator | 1.24 | 0.0407 | 9.51 | 9.53 | 9.41 | 9.22 | 9.16 | 9.15 | Cytoplasm | other |
| 1443970_at | *Ntrk3* | neurotrophic tyrosine kinase, receptor, type 3 | 1.28 | 0.0411 | 4.8 | 4.78 | 4.62 | 4.39 | 4.27 | 4.48 | Plasma Membrane | kinase |
| 1449796_at | *Prph* | peripherin | 1.28 | 0.0415 | 5.95 | 5.94 | 5.96 | 5.65 | 5.59 | 5.55 | Plasma Membrane | other |
| 1439824_at | *Chm* | choroideremia (Rab escort protein 1) | 1.30 | 0.0407 | 9.21 | 8.98 | 9.02 | 8.73 | 8.69 | 8.66 | Cytoplasm | enzyme |
| 1420349_at | *Ptgfr* | prostaglandin F receptor (FP) | 1.32 | 0.0408 | 4.91 | 4.87 | 4.78 | 4.44 | 4.35 | 4.58 | Plasma Membrane | G-protein coupled receptor |
| 1438532_at | *Hmcn1* | hemicentin 1 | 1.32 | 0.0407 | 7.99 | 7.92 | 7.85 | 7.62 | 7.54 | 7.4 | Extracellular Space | other |
| 1443749_x_at | *Slc1a3* | solute carrier family 1 (glial high affinity glutamate transporter), member 3 | 1.36 | 0.0407 | 9.71 | 9.46 | 9.68 | 9.3 | 9.07 | 9.14 | Plasma Membrane | transporter |
| 1424208_at | *Ptger4* | prostaglandin E receptor 4 (subtype EP4) | 1.47 | 0.0495 | 6.5 | 6.94 | 6.31 | 6.12 | 5.97 | 5.98 | Plasma Membrane | G-protein coupled receptor |

The number in the column heading (Table S3) represents the mouse identity used in the microarray analysis.
